# Influence of variant-specific mutations, temperature and pH on conformations of a large set of SARS-CoV-2 spike trimer vaccine antigen candidates

**DOI:** 10.1038/s41598-023-43661-2

**Published:** 2023-10-01

**Authors:** Matthew Stuible, Joseph D. Schrag, Joey Sheff, Daria Zoubchenok, Simon Lord-Dufour, Brian Cass, Denis L’Abbé, Alex Pelletier, Martin A. Rossotti, Jamshid Tanha, Christian Gervais, Roger Maurice, Majida El Bakkouri, Mauro Acchione, Yves Durocher

**Affiliations:** 1 Human Health Therapeutics Research Centre, National Research Council Canada, 6100 Royalmount Avenue, Montreal, QC H4P 2R2 Canada; 2 Human Health Therapeutics Research Centre, National Research Council Canada, Ottawa, ON Canada

**Keywords:** Biochemistry, Biotechnology, Drug discovery, Molecular biology, Structural biology

## Abstract

SARS-CoV-2 subunit vaccines continue to be the focus of intense clinical development worldwide. Protein antigens in these vaccines most commonly consist of the spike ectodomain fused to a heterologous trimerization sequence, designed to mimic the compact, prefusion conformation of the spike on the virus surface. Since 2020, we have produced dozens of such constructs in CHO cells, consisting of spike variants with different mutations fused to different trimerization sequences. This set of constructs displayed notable conformational heterogeneity, with two distinct trimer species consistently detected by analytical size exclusion chromatography. A recent report showed that spike ectodomain fusion constructs can adopt an alternative trimer conformation consisting of loosely associated ectodomain protomers. Here, we applied multiple biophysical and immunological techniques to demonstrate that this alternative conformation is formed to a significant extent by several SARS-CoV-2 variant spike proteins. We have also examined the influence of temperature and pH, which can induce inter-conversion of the two forms. The substantial structural differences between these trimer types may impact their performance as vaccine antigens.

## Introduction

Whether introduced into vaccine recipients via a genetically-encoded expression vector (mRNA, DNA or recombinant virus) or as a purified antigen (soluble protein, VLP/nanoparticle, etc.), the spike protein of the SARS-CoV-2 virus remains the antigen of choice for COVID-19 vaccine development. The SARS-CoV-2 spike consists of a large N-terminal ectodomain with a C-terminal transmembrane domain securing it to the virus envelope, where it adopts its characteristic trimeric conformation^1–3^. The receptor binding domain (RBD) of the spike binds to ACE2 on the host cell surface and initiates the process of fusion and cell entry, leading to viral replication. The effectiveness of COVID-19 vaccines correlates with their ability to induce anti-spike neutralizing antibodies that block this interaction^4^; while most target the RBD directly, anti-NTD and -S2 neutralizing antibodies have also been reported^5,6^.

While mRNA- and viral vector-based products were the first to be approved as COVID-19 vaccines in North America and Europe, several protein subunit vaccines have now been approved or are in late-stage clinical trials. Although some are based on the RBD or other fragments of the spike protein, most protein subunit vaccine candidates are designed around presentation of the complete spike ectodomain. The Nuvaxovid vaccine (Novavax) includes the full-length spike protein antigen with its natural transmembrane domain^7^. Alternatively, soluble spike ectodomain constructs that mimic the native spike structure can be produced by replacing the transmembrane domain and C-terminal tail with a heterologous trimerization sequence, such as the human resistin or the bacteriophage T4 foldon or human collagen type I (α) domains^1,8,9^. Multiple protein subunit vaccines based on this design have reached clinical testing^9,10^, including recently-reported phase 3 trials by Clover Biopharmaceuticals^11^ and Sanofi/GSK^12^.

The structures and biophysical properties of trimeric spike ectodomain constructs have been characterized extensively. Since the beginning of the pandemic, numerous cryo-EM studies have documented the flexibility of ectodomain protomers to adopt RBD-up and -down conformations, the proportions of which are variant-dependent and dictate the capacity of the spike to bind ACE2^3^. Interestingly, it was recognized only recently that such constructs can also adopt an alternative conformation that is quite distinct from the RBD-up and -down prefusion forms: hydrogen–deuterium exchange (HDX) mass spectrometry analysis by Costello et al. revealed an “open” trimeric conformation consisting of loosely-associated ectodomain protomers in which inter-protomer interfaces are more exposed^13^. The ability of protein subunit vaccine antigens to adopt this conformation may impact immunogenicity, as epitopes normally inaccessible in conventional trimer structures may be exposed in this alternative conformation^13^.

Since early 2020, our group has produced a large variety of soluble, trimeric SARS-CoV-2 spike protein constructs in CHO cells to support development of COVID-19 vaccines as well as diagnostic and serological assays^8,14–19^; these include over a dozen naturally-occurring and engineered spike sequence variants produced as fusions to bacteriophage T4 foldon or human resistin. While different eukaryotic host cell lines have been used to produce recombinant spike trimers, including insect cells for some approved vaccines (Nuvaxovid^7^ and VidPrevtyn Beta^12^) and human HEK293 cells for many structural studies, we showed recently that CHO cells give considerably higher spike yields than HEK293 cells^8^. Combined with the ability of CHO cells, unlike insect cells, to generate proteins with human-like glycosylation^20^, we believe that CHO cells would be a preferable host for spike subunit vaccine manufacturing^21^.

Routine evaluation of these recombinant spike constructs by analytical size exclusion chromatography-multi-angle light scattering (SEC-MALS) revealed that two trimer species could often be resolved; we refer to these as ‘trimer 1’ and ‘trimer 2’. We applied a number of additional orthogonal biophysical techniques to examine how different factors influence the preference of different constructs to adopt each of these forms. We also identify antibodies that bind preferentially to the more compact trimer 2 species. Our results support a model of reversible interconversion between compact (trimer 2) and loosely-associated (trimer 1) conformations that is impacted by temperature, pH and variant-specific mutations. These observations on vaccine-relevant constructs are discussed within the broader context of potential impact on protein-subunit vaccine manufacturability, efficacy, and stability.

## Results

### Protein production

Soluble spike constructs were purified from supernatants of transiently or stably transfected CHO cells; a summary of all constructs tested in this study is provided in Supplementary Table 1. Purified proteins separated by SDS-PAGE and stained with Coomassie Blue are shown in Supplementary Fig. 1. All variants show high purity with only traces of other bands. For some variants, particularly those with the foldon trimerization domain, high molecular weight bands are detected, which are reducible in buffer containing DTT; these bands are detected by anti-spike western blotting (not shown) and may be the result of low-level intermolecular disulfide bonds or some other disulfide-mediated oligomerization process. The D614G variant (S(Ref-D614G)-R) also shows traces of low molecular weight bands. Notably, the Omicron (S(BA1)-R) preparation under reducing conditions shows conspicuous lower molecular weight bands that we have confirmed by mass spectrometry to be spike protein fragments, likely the result of cleavage at a site (or sites) distinct from the S1/S2 furin site which is mutated in all of the constructs we have produced.

### Analytical SEC

**Figure 1:**
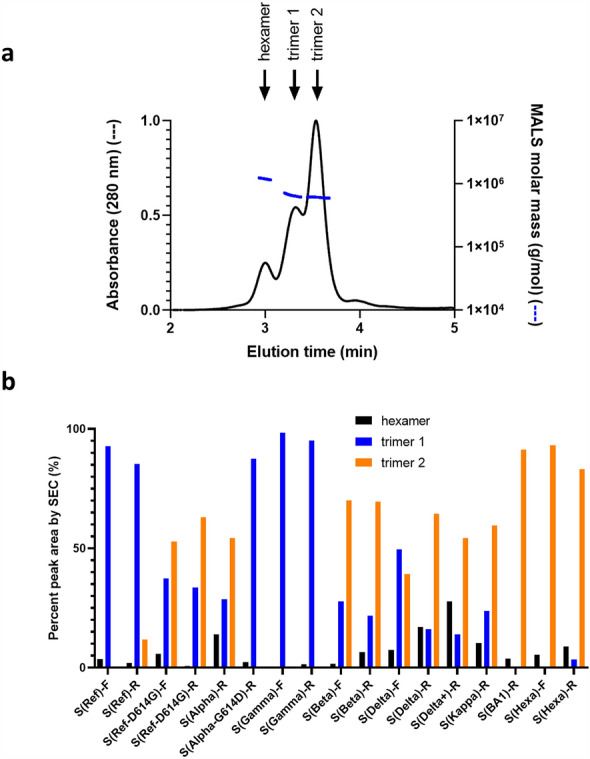
SARS-CoV-2 spike protein variants display variable levels of two trimer peaks by analytical size exclusion chromatography (SEC). (**a**) Example of an A_280_ SEC profile of Alpha variant spike ectodomain fused to resistin (S(Alpha)-R) showing peaks with molar masses consistent with hexamers and two distinct trimer species. (**b**) Distribution of trimer and hexamer SEC peaks for a selection of spike variants in DPBS at pH 7.8.

Analytical SEC profiles were highly variable for different spike constructs in our default formulation buffer (DPBS, pH 7.8), with the bulk of the peak area distributed among three peaks: one whose molar mass assessed by MALS is consistent with hexamers (likely dimers of trimers^7^) and two peaks with masses consistent with spike trimers. An example of a SEC-MALS profile showing the 3 peaks is shown in Fig. 1a. The ability to resolve these two trimer types by SEC was suggestive of substantial conformational differences between the two forms: a more extended, ‘open’ conformation for the faster-eluting trimer 1 and a more compactly folded structure for the slower-eluting trimer 2.

The relative SEC peak areas for a panel of spike constructs is shown in Fig. 1b and Supplementary Tables 2 and 3. The reference (Wuhan) construct fused to resistin (S(Ref)-R) consists primarily of trimer 1, with a small amount trimer 2 detected as a late-eluting shoulder or minor second peak. Trimer 1 is also predominant for the Gamma variant (S(Gamma)-R). The Beta variant spike fused to resistin (S(Beta)-R) exhibits mostly trimer 2 with a small amount of trimer 1. The Omicron spike variant (S(BA1)-R) consists almost exclusively of trimer 2, as does HexaPro (S(Hexa)-R), which contains 4 additional, engineered proline mutations that further stabilize the prefusion spike protein compared to the 2P mutations present in all other constructs^22^. For several variants, the same spike sequences were also produced as foldon fusions. Similar to resistin fusions, the reference-strain and Gamma variant foldon fusions (S(Ref)-F and S(Gamma)-F) consist predominantly of trimer 1, whereas the Beta and Delta variants (S(Beta)-F and S(Delta)-F) show a mix of trimers 1 and 2. The proportions of trimer 1 *vs* trimer 2 for foldon constructs are, for most variants, similar to those observed for the resistin constructs. The lone exception is the Delta variant, for which the resistin construct exhibits higher trimer 2 content than the foldon construct.

Most known spike variants contain the well-studied D614G mutation, one of the first problematic mutations to be identified circulating in the population^23^. As shown in Fig. 1b, mutation of D614 to G in the context of the reference strain spike sequence (S(Ref-D614G)-F and -R) increases the proportion of trimer 2 regardless of the trimerization domain used. The Alpha variant, whose distribution is predominantly trimer 2, becomes predominantly trimer 1 upon reversion of G614 to D (S(Alpha-G614D)-R). The presence of D at amino acid 614 is, thus, associated with a predominance of trimer 1. The Gamma variant (S(Gamma)-F and -R) was the only variant containing a G at position 614 that resembled the reference strain with a distribution favoring trimer 1. Given that the amino acid identity at position 614 in the reference strain, Alpha, and Gamma variants is by itself insufficient to effect complete conversion to one or the other trimeric forms, additional mutations present in the variants must also contribute significantly to the distribution of conformations.

### DSC and DSF

**Figure 2:**
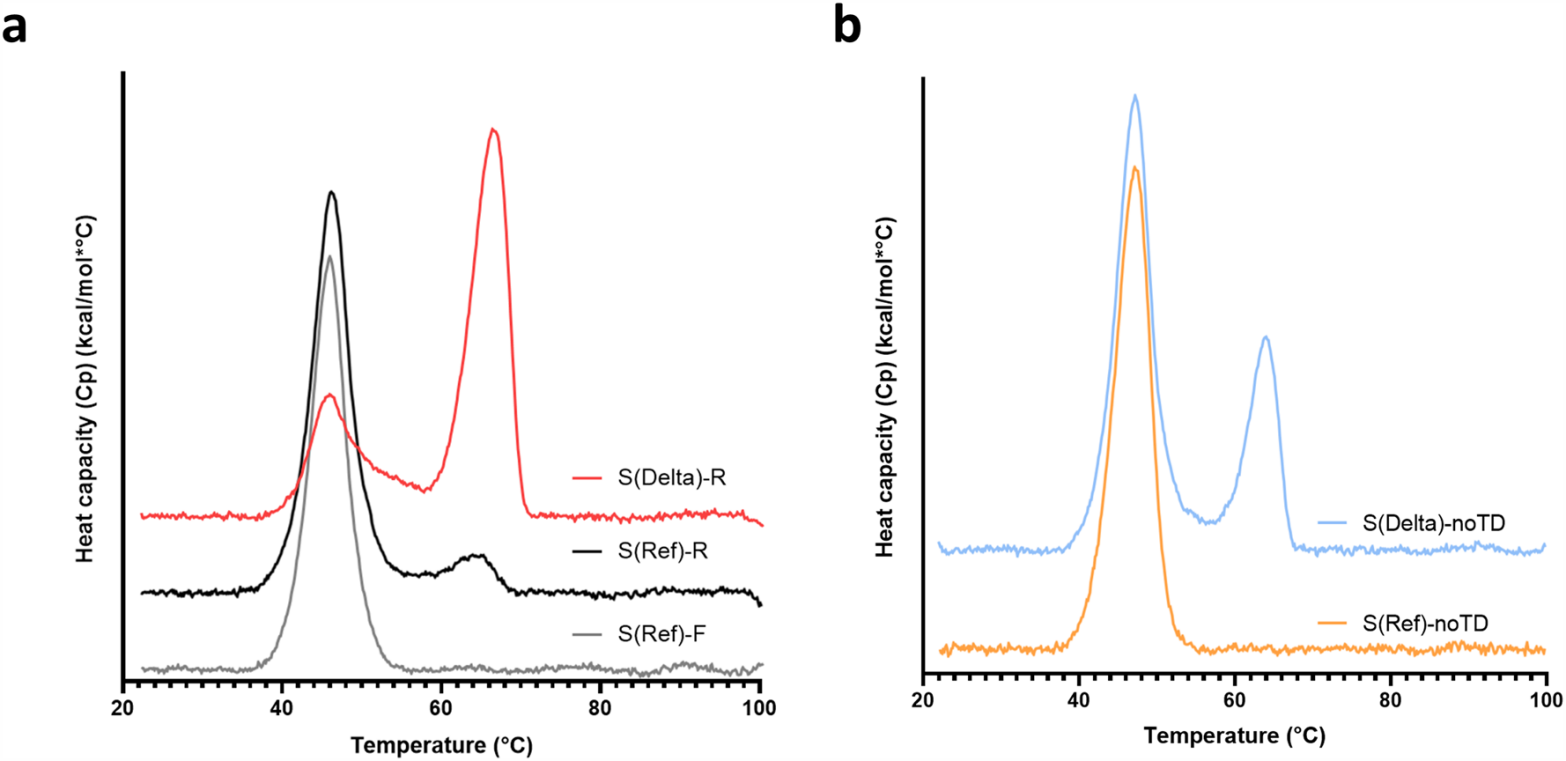
A high-temperature melting transition by DSC correlates with the presence of trimer 2 species for different SARS-CoV-2 spike constructs. (**a**) Compared to the S(Ref)-F construct that exhibits a single, low-temperature melting transition, a high-temperature transition is detectable for S(Ref)-R and much more prominent for S(Delta)-R. (**b**) S(Delta)-noTD, expressed without a heterologous trimerization domain, is purified as a mix of monomeric and trimeric species and exhibits a high-temperature melting transition similar to trimeric species containing trimer 2. S(Ref)-noTD produced in the same way is monomeric and exhibits only the low-temperature transition.

Characterization by differential scanning calorimetry (DSC) revealed that the presence of trimer 2 by UPLC-SEC correlates with a distinct and well-resolved unfolding transition in DSC melting curves. Spike preparations containing trimer 2, such as the Delta resistin fusion shown in Fig. 2a, show a characteristic unfolding transition at 63 ± 2 °C in addition to the transition at 47 ± 2 °C shared by all spike ectodomain constructs. For trimeric spike preparations containing lower levels of trimer 2 (S(Ref)-R and -F, Fig. 2a), the high-T_m_ transition is less pronounced or absent. As shown in Fig. 2b, a mostly monomeric preparation of reference-strain spike without a heterologous trimerization domain (S(Ref)-noTD^8^) displays a low-temperature unfolding transition similar to that of the trimer 1. Notably, the corresponding Delta spike construct without trimerization domain (S(Delta)-noTD) is 30–50% trimeric, with the remainder mostly monomeric. The self-assembled (ie. not trimerization domain-mediated) trimers formed by this construct, analyzed as a mixture with the monomeric form, show the presence of the high-T_m_ melting transition by DSC characteristic of trimer 2 (Fig. 2b). As shown in Supplementary Fig. 2, several spike preparations were also analyzed, in DPBS at pH 7.8, by differential scanning fluorimetry (DSF); this technique monitors protein unfolding in a temperature gradient using a fluorescent probe. The DSF results are consistent with the DSC results. The DSF is showing two main transitions across the tested variants, a lower temperature melting transition at 43.9 ± 1.6 °C and a higher temperature melting transition at 63.5 ± 2.4 °C. As observed with DSC, the high-Tm transition is more pronounced for preparations containing trimer 2. These results do not permit assignment of different melting transitions to specific features of the analyzed proteins, but the significantly higher thermal stability of trimer 2 suggests the presence of stabilizing inter-protomer interactions not present in trimer 1 or in monomeric spike constructs.

### HDX mass spectrometry

HDX mass spectrometry was deployed to probe the conformational differences between the trimerization states. This method monitors hydrogen–deuterium exchange rates as an indicator of solvent accessibility throughout the spike protein sequence. Faster exchange rates suggest greater solvent accessibility, whereas slower exchange occurs in more buried residues. A summary of HDX experimental parameters and results are shown in Supplementary Table HDX-1 and 2 and Supplementary Fig. 3. It was not possible to assign differential HDX values for spike protein regions mutated in SARS-CoV-2 variants since intrinsic amide exchange rates are influenced by primary structure^24^, leading to construct-dependent gaps in sequence. This was compounded by the extensive N-linked glycosylation of the spike protein.

**Figure 3:**
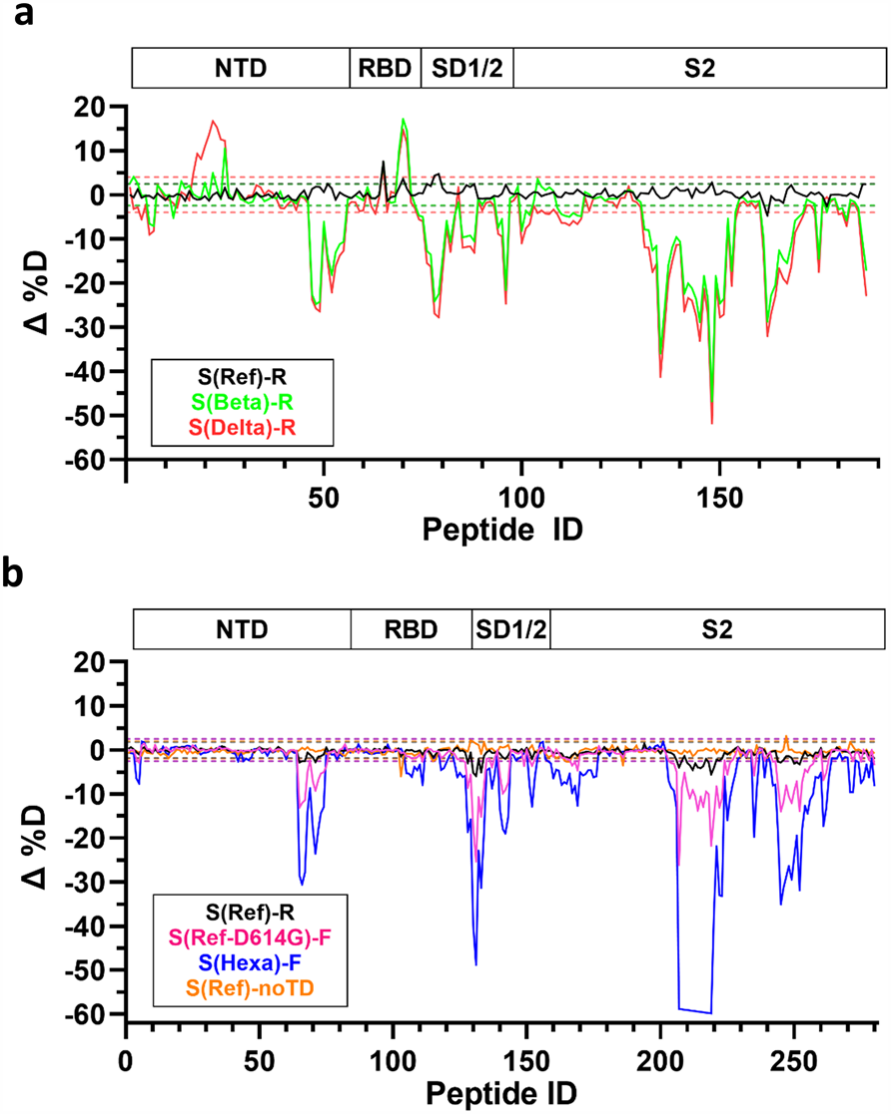
HDX-MS Woods plots. Differential normalized HDX relative to S(Ref)-F (trimer 1) at a single representative HDX time point (60 min). Δ%D is plotted as a function of peptide ID (see Table HDX-S2 for a full list of peptides). S(Beta)-R and S(Delta)-R (green and red, respectively) are plotted in (**a**), while S(Hexa)-F and S(Ref)-noTD (blue and red, respectively) are plotted in (**b**). S(Ref)-R (black) is included in both plots as a reference. Data in (**a**) and (**b**) were collected in triplicate in two separate batches. Dashed lines represent ± 3× pooled SD for each unique state. Δ %D measurements outside the dashed lines demonstrate either significantly reduced exchange (− Δ %D) or increased exchange (+ Δ %D) based on a 1-p value of 0.98. Key structural domains are indicated above the Woods plots.

**Figure 4:**
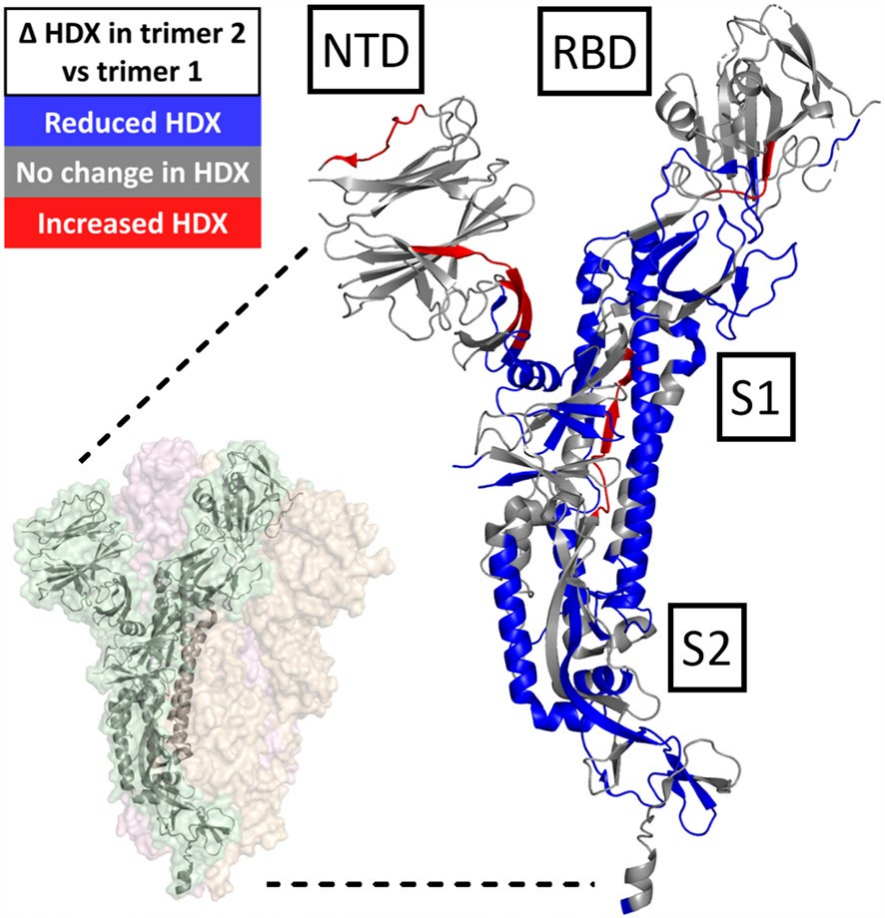
Projection of HDX onto 3-D structure. Differential normalized HDX of S(Hexa)-R (trimer 2) relative to S(Ref)-F (trimer 1) is projected onto a single protomer, where residues with significantly reduced or increased exchange in at least one labelling time point are shown in blue and red, respectively. The full spike trimer is inset as a reference (PDB 6VXX). Significance is based on a 1-p value of 0.98 and 3× pooled standard deviation.

Constructs containing mostly trimer 2 (eg. Delta, Beta and HexaPro resistin or foldon fusions) show widespread changes in solvent accessibility compared to the predominantly trimer 1 reference strain- foldon fusion used as a reference in this analysis (Fig. 3). A representative trimer 2 dataset (S(Hexa)-R) is shown on a three-dimensional structure of the spike trimer (Fig. 4). Furthermore, the magnitude of HDX shifts increases proportionally to the trimer 2 population. Notably, regions for which exchange rates decrease for trimer 2 preparations are concentrated in the S2 domain, which hosts a significant portion of the inter-protomer interface. Exchange rate decreases also occur at other sites of inter-protomer interactions outside of the S2 domain, particularly at the C-terminus of the NTD. A twisting motion centered on this region has been proposed to disrupt the strength of the protomer interactions^25^. Conformational dynamics within the RBD remained largely unaltered with the exception of decreased exchange (up to ΔD = 50%) over the entire SD1 region, which acts as a dynamic hinge for RBD transitions between up/down states^26^. In constructs for which trimer 2 dominates, we also observed discrete regions where solvent accessibility is increased: across residues Y265-F275 (NTD), P621-Y636 (unstructured loop in SD2), and P728-K737 (S2) (Supplementary Fig. 3). Additionally, increased exchange in residues N487-Y495 (RBD) observed only for Beta and Delta variants (Fig. 3a**,** green and red) is consistent with disruption of the E484 interaction with F490^27^, and likely is not a consequence of the structural transition from trimer 1 to 2. Similarly, a destabilization in residues F92-K113 (NTD) was unique to the Beta and Delta variants. Importantly, the monomeric reference-strain spike expressed without fusion to a trimerization sequence exhibits an HDX profile highly similar to the trimeric reference-strain spike-foldon fusion consisting of trimer 1 only (Fig. 3b, orange). These results suggest that, in the trimer 1 conformation, individual spike protomers are loosely associated, with minimal inter-protomer contact, while trimer 2 is a more compactly folded conformation.

### Effects of temperature on conformation

Prefusion-stabilized SARS-CoV-2 spike trimers comparable to the S(Ref)-F construct used in the current study have been reported to be destabilized/denatured by prolonged incubation at 4 °C, as indicated by negative stain EM and DSC^28,29^. This destabilization is reversible with subsequent incubation at 37 °C or by lowering formulation buffer pH^13,28,29^. Costello et al. demonstrated by HDX that the low-temperature, destabilized form of the spike corresponds to the open-trimer conformation^13^. We have observed similar temperature effects as well: trimer 1 content for many variants was observed to increase with storage at 4 °C for 2, 14 and 24 weeks at pH 7.8 but not at pH 5.5 (24-week results shown in Supplementary Fig. 4), although complete conversion to trimer 1 was not observed, even after 24 weeks. This result is not consistent with the results of Olia et al. which indicated that 30 days at 4 °C is sufficient to cause denaturation of the spike^29^. Although the spike protein construct used in the Olia et al. study is highly similar to our S(Ref)-F construct, the production host (HEK *vs* CHO) and purification method were different; host-dependent differences in glycosylation are one potential explanation for the differences in spike stability between the two studies, although we do not have results to support this. Conversely, incubation at 37 °C for 1 or 2 days was insufficient to completely convert trimer 1 to trimer 2. Incubation for 2 days at 37 °C after 24 weeks at 4 °C significantly increased trimer 2 content (Supplementary Fig. 5), indicating at least partial reversibility of the temperature-dependent conformational change observed at pH 7.8.

### Effects of pH on conformation

**Figure 5:**
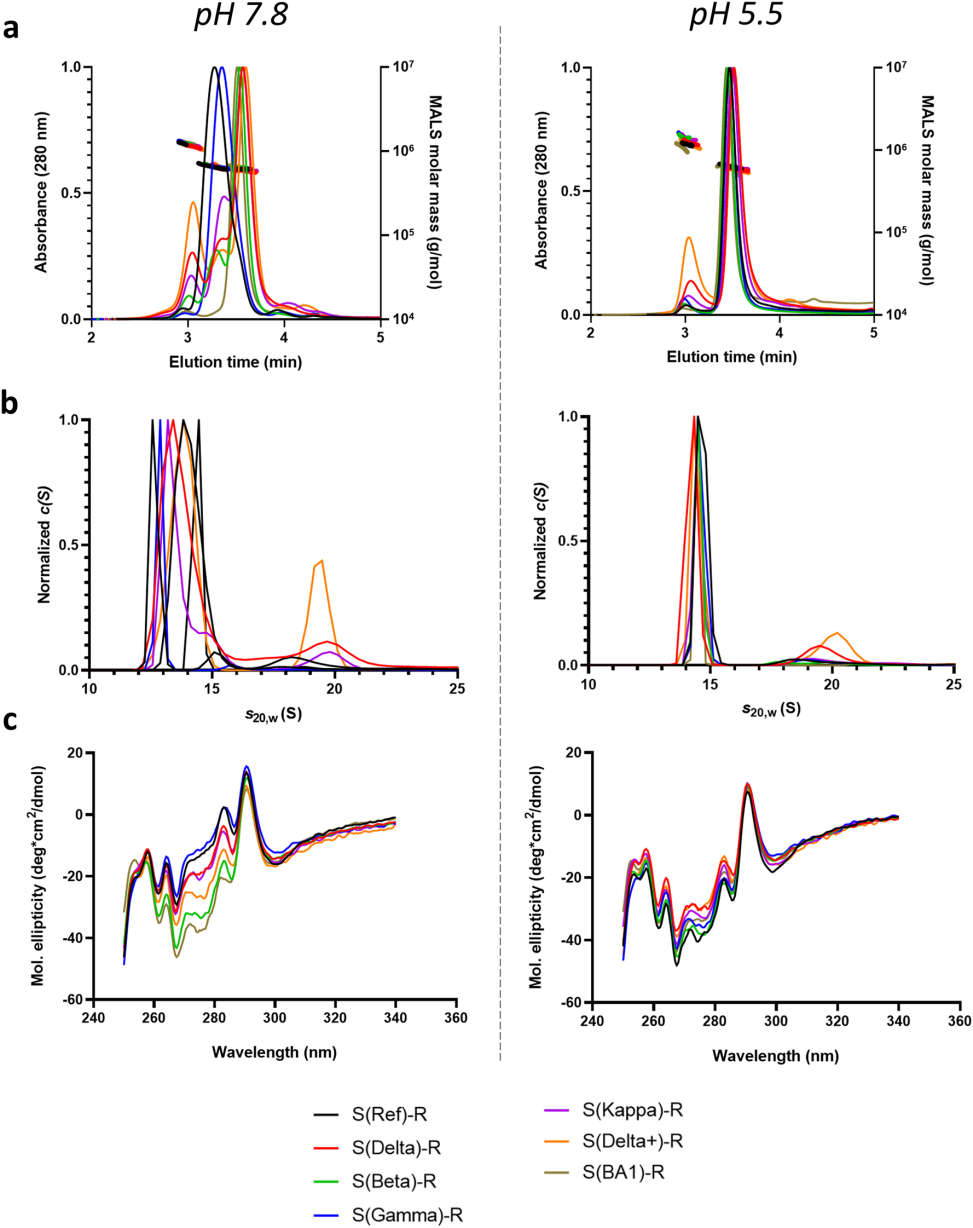
Conformational variability of spike-resistin fusion constructs is reduced at low pH. UPLC-SEC (**a**), SV-AUC (**b**) and near-UV CD (**c**) all demonstrate that conformational variability observed at pH 7.8 is greatly reduced at pH 5.5. All data shown are for resistin fusion constructs.

In our hands, the effect of pH on spike conformation is more pronounced than the temperature effects. As shown in Fig. 5 and Supplementary Figs. 6 and 7, with pH adjustment from 7.8 to 5.5 all spike constructs are converted to a conformation with a trimer 2-like SEC profile. In contrast to pH 7.8, storage of spike constructs at pH 5.5 for 24 weeks at 4 °C resulted in little change in trimer 2 content as assessed by SEC, although in some cases hexamer content increased slightly (Supplementary Fig. 4B). Even for variants which had converted almost completely to trimer 1 after 24 weeks of storage at pH 7.8 at 4 °C, buffer exchange to pH 5.5 converted almost all trimer 1 to trimer 2 (Supplementary Fig. 8A), indicating the reversibility of pH-induced changes in conformation. Less pronounced changes in conformation were detected after buffer exchange to pH 7.8 for samples stored for 24 weeks at pH 5.5 (Supplementary Fig. 8B), likely reflecting slower kinetics of conversion of trimer 2 to trimer 1.

### Analytical ultracentrifugation

As shown in Fig. 5b, Supplementary Fig. 7B and Supplementary Tables 2 and 3, analytical ultracentrifugation (AUC) sedimentation velocity analysis is consistent with the SEC-MALS results: at pH 7.8 in phosphate buffer, those constructs that favor trimer 1 (e.g. S(Ref) and S(Gamma) fused to foldon or resistin) sediment at 12.5–12.8 S, whereas those that adopt predominantly the trimer 2 conformation (S(BA1)-R) sediment near 14.4 S, consistent with a more compact conformation. Those variants that show mixtures of trimers 1 and 2 (S(Beta), S(Delta), S(Delta+) and S(Kappa)) show broader distributions with variable main peak S values in the 13.5–14 S range. In contrast, at pH 5.5 all constructs sediment at 14.2–14.5 S, consistent with a more compact conformation. As was observed by SEC-MALS, the trimerization domain employed has for most variants little impact on AUC profiles (Fig. 5b and Supplementary Fig. 6B). The lone exception is the Delta variant, for which the construct with the foldon trimerization domain (S(Delta)-F) sedimented more similarly to the reference and Gamma variants compared to the Delta resistin construct (S(Delta)-R)). The HexaPro foldon construct (S(Hexa)-F) at low pH sediments slightly slower than other foldon variants.

### Circular dichroism

Circular dichroism (CD) analysis was employed to further gauge the conformational changes inferred by SEC-MALS and AUC. The far UV CD spectra are minimally affected by pH changes, indicating that any conformational changes do not involve major alterations of secondary structural elements (results for S-resistin fusions shown in Supplementary Fig. 9). In the near UV at neutral pH, the CD spectra show significant differences between variants, whereas at pH 5.5 the spectra become much more similar (Fig. 5c and Supplementary Fig. 7C). The extent of the spectral changes correlates with the predominance of trimer 1: those variants for which trimer 1 predominates at pH 7.8 show the largest spectral changes upon lowering pH (Supplementary Tables 2 and 3). The changes as a function of pH are greatest in the 275–285 nm region, where the major contribution to the signal is from tyrosine residues. pH-induced changes in the near UV CD spectra are observed independently of which trimerization domain is used. The D614G mutation alone induces some shift in the CD spectrum, but additional shifts are induced upon lowering the pH (Supplementary Fig. 7C).

### Conformation-dependent binding of anti-spike V_H_Hs by ELISA

**Figure 6:**
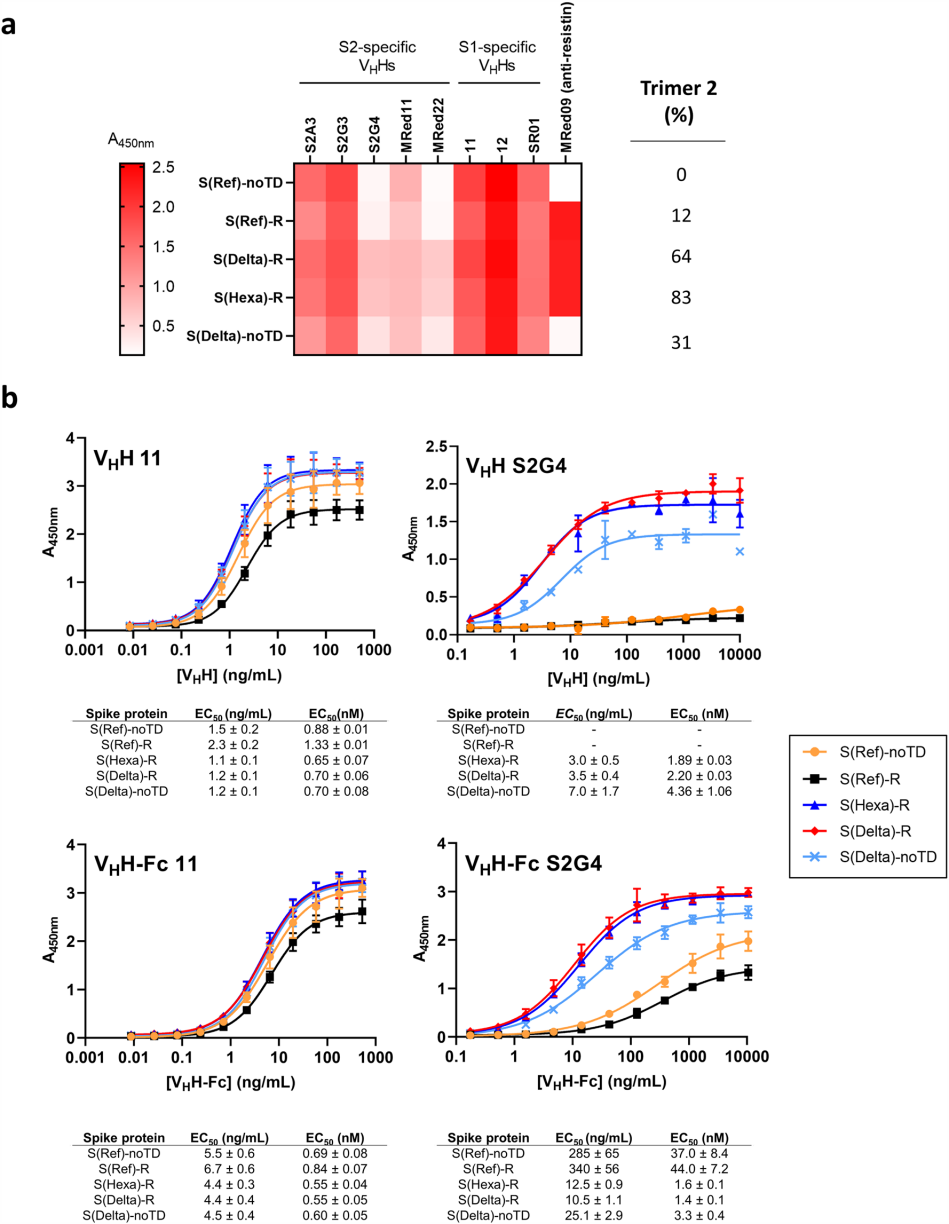
Anti-spike S2 subunit V_H_Hs S2G4 and MRed22 bind preferentially to spike constructs containing trimer 2. (**a**) Single-concentration screening of V_H_Hs for binding to different spike preparations by ELISA. Microwell plates were coated with the indicated spike antigens binding to a single concentrations of different V_H_H-Fcs was assessed. S2A3, S2G3, S2G4, MRed11, and MRed22 were characterized previously as S2-specific binders, while 11, 12 and SR01 bind different epitopes on the S1 subunit. MRed09 binds the resistin trimerization sequence. Shades of red indicate the absorbance measured by ELISA for the different conditions. (**b**) Dose–response analysis of V_H_H 11 and S2G4 binding to spike preparations with varying proportions of trimer 1 and trimer 2. S2G4 binds with lower EC_50_ to spike preparations consisting mostly of trimer 2 compared to preparations consisting mostly of trimer 1. In contrast, V_H_H 11 binds similarly to both forms. ELISAs were performed with both dimeric, Fc-fused V_H_Hs (top panels) and monomeric, biotinylated V_H_Hs (bottom panels). EC_50_s are averages of three technical replicates and error bars correspond to standard deviation.

Based on the substantial conformational differences between trimer 1 and 2 inferred by all of these orthogonal analytical methods, we expected that certain antibody epitopes of the spike protein that are exposed in trimer 1 could be masked in trimer 2. Indeed, Costello et al. reported one such antibody with specificity for trimer 1 *vs* trimer 2^13^. Conversely, conformational epitopes present at sites of inter-protomer contacts might only be present in trimer 2 samples. We recently described a collection of novel V_H_Hs with a diverse set of epitopes encompassing the full ectodomain of the SARS-CoV-2 spike^16^. We screened several of these V_H_Hs (in V_H_H-Fc format) by ELISA for binding to spike constructs consisting primarily of trimer 1 or trimer 2: while most V_H_H-Fcs, including both S1 and S2 subunit-specific antibodies, bound similarly to the two forms, we found two (S2G4 and MRed22) that bound preferentially to spike preparations consisting mostly of trimer 2 (eg. S(Delta)-R and S(Hexa)-R). A heat map illustrating ELISA results at a single concentration with multiple anti-spike V_H_H-Fcs (as well as the anti-resistin V_H_H-Fc MRed09) is shown in Fig. 6a. For S2G4 (selective trimer 2 binder) and V_H_H 11 (non-selective spike binder), dose–response analyses were performed to estimate EC_50_ values using monomeric and dimeric (Fc-fused) V_H_H formats. As shown in Fig. 6b, V_H_H 11 and V_H_H 11-Fc bind all spike preparations with EC_50_s of 0.6–1.3 nM. In contrast, S2G4 and S2G4-Fc bind predominantly trimer 2 spike preparations with similarly low EC_50_s (1.4–2.2 nM) but less well to preparations consisting mostly of trimer 1 (S(Ref)-R) or monomeric spike (S(Ref)-noTD) (EC_50_ > 37 nM). S(Delta)-noTD, which is a mixture of trimer 2-like and monomeric species, binds with intermediate EC_50_. Overall, the dose–response results are similar for monomeric and dimeric V_H_H formats, with somewhat greater differences in binding to the two conformations exhibited by the monomeric V_H_Hs.

## Discussion

Given the structural flexibility of the spike, it is important to consider whether our observations could be explained by the presence of other previously-described spike conformations. The prefusion to post-fusion conversion that occurs upon virus binding to host cells is a major structural rearrangement that changes the biophysical properties of the spike protein considerably. However, previous characterization by cryo-EM of spike-foldon fusions highly similar to those used in the current study found no evidence of trimers adopting a post-fusion conformation^30^. Also, the post-fusion conformation (like the conventional prefusion conformation) retains tight interactions between spike S2 protomers (although different than in prefusion trimers)^31^. As suggested by the similarity in HDX for reference-strain spike monomers and trimer 1 species, such interactions are largely absent in the trimer 1 conformation we have observed, making a post-fusion conformation unlikely to explain our observations.

Prefusion spike trimers can also adopt well-characterized conformations with individual RBDs in “up” and “down” positions (these are sometimes referred to as “open” and “closed” prefusion conformations). Zhou et al. have demonstrated these domain movements to be pH-dependent, but the RBD-up and –down conformations have little impact on the structure of the S2 region^32^. Whether simple RBD movements could alter the globular structure of trimers sufficiently to produce the SEC elution time shifts we observed is unclear. However, RBD movements alone do not explain the substantial differences in S2 domain-concentrated inter-protomer interactions we detected by HDX. Also, the differences in binding we observed for S2-directed VHHs to trimer 2 *vs* trimer 1 are unlikely to be directly caused by differences in RBD positioning only. It is therefore unlikely that RBD movements can account for all of the biophysical and immunological differences between trimers 1 and 2 that we have observed.

Temperature and pH sensitivity of soluble spike preparations has been reported^13,28,32^. The presence of well-ordered and poorly ordered spike trimers was visualized by negative stain EM (NS-EM) and cryo-EM^28,29,32^. Zhou et al. reported that for a reference strain spike-foldon construct, the proportion of well-ordered structures observable by NS-EM is greatly affected by pH, with well-formed trimers rarely visible after storage in PBS but readily detectable at pH 5.5 and lower^32^. A similar disordered state was observed by NS-EM after prolonged incubation of spike trimers at 4 °C^28^, a change which can be reversed by pH adjustment to 4.0–5.5^29^. Thermal stability and antibody binding were impacted by both temperature and pH induced conformational changes^28,29^. Based on HDX analysis Costello et al. proposed that low temperature or ACE2 binding induces a similar conversion to a conformation with reduced inter-protomer association of S2 regions^13^.

**Figure 7:**
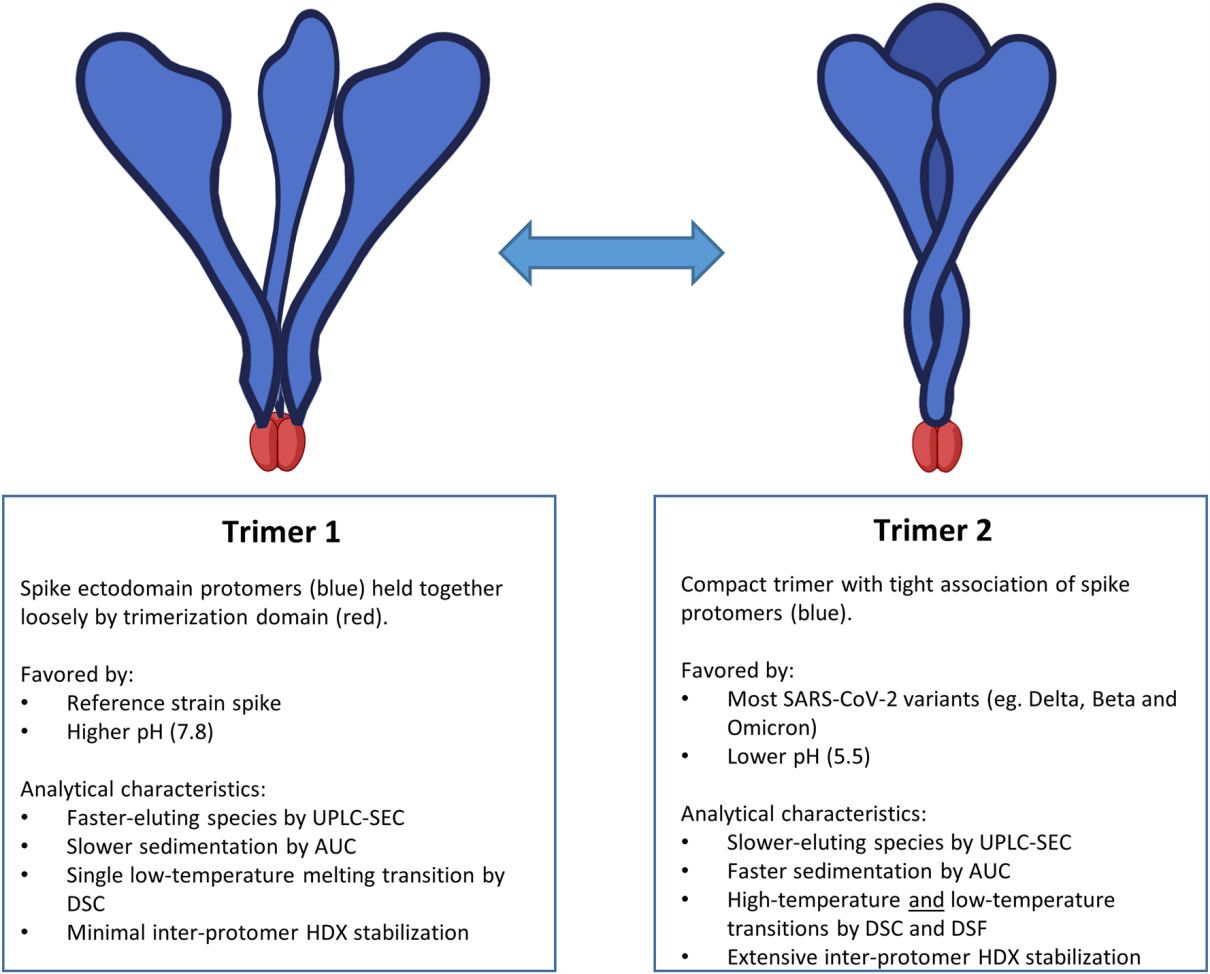
Spike trimer models. Trimer conformations adopted by SARS-CoV-2 spike ectodomain-foldon and resistin fusions supported by the current study.

The SEC, AUC, CD, DSC, DSF, HDX and immunological results we have presented are consistent with the model illustrated in Fig. 7. Trimer 1, which has properties consistent with the open conformation recently described by Costello et al.^13^, is dominant for reference strain constructs fused to either foldon or resistin trimerization domains when formulated at pH 7.8. Our HDX and DSC results demonstrate that monomeric spike protein is essentially indistinguishable from the trimer 1 species formed when the same sequence is fused to foldon or resistin, indicating that the trimer 1 conformation consists of three spike ectodomain protomers held together loosely with limited S2 inter-protomer interactions. In contrast, many spike variant ectodomains fused to the same trimerization sequences form more compact and thermally stable trimers (trimer 2 conformation) at the same pH (7.8). At lower pH (5.5), a trimer 2-like conformation is favored for all spike constructs we have tested. There are subtle differences in the biophysical properties of trimer 2 species at pH 7.8 *vs* the conformation adopted by all constructs at pH 5.5 (eg. near-UV CD, AUC); these are likely the result of additional effects of pH on spike structure that we have not characterized here. Trimer 2 preparations exhibit later elution times by UPLC-SEC and faster sedimentation by AUC. In our hands, the presence of trimer 2 as indicated by UPLC-SEC always corresponds to the presence of higher-temperature melting transitions by DSC, which are likely the result of the extensive inter-protomer contacts observed for trimer 2 preparations by HDX. None of the analyses we have performed indicate any consistent differences in trimers formed by foldon *vs* resistin trimerization sequences.

In reference-strain spike trimers, D614 is involved in inter-protomer hydrogen bonding, which is disrupted in variants containing the D614G mutation^33^. Another effect of D614G is to promote adoption of the RBD-up conformation^30,33,34^. Both the reduced inter-protomer interactions and the preference for the RBD-up *vs* -down conformation caused by D614G would intuitively favor the loosely-associated trimer 1 conformation; however, our results clearly indicate that D614G has the opposite effect, promoting the more compact trimer 2 form. This observation is not easily explained by current literature and supports the distinction between the “open” RBD-up conformation and the trimer 1 species we have characterized. Notably, D614G by itself is insufficient to shift the conformation completely to trimer 2. Therefore, other mutations present in different variants must also promote adoption of the more compactly assembled trimer 2 conformation. Interestingly, a lysine residue, K986, implicated in formation of an inter-protomer salt bridge in spike trimers^35^, is the site of one of the prefusion-stabilizing proline mutations present in all of our spike constructs (as well as most S protein subunit vaccine candidates). It is possible that this specific mutation may artificially favor more open spike conformations relative to wild-type spike sequences.

A direct comparison of the performance of spike preparations favoring trimer 1 *vs* trimer 2 as vaccine antigens is difficult, as mutations favoring either conformation may affect the spike structure in other ways as well. Our results as well as those of Edwards et al.^28^ and Costello et al.^13^ demonstrate the potential for spike vaccine-induced generation of antibodies against conformational epitopes present only in trimer 2 as well as other epitopes exposed only in trimer 1. If, as proposed^13^, a trimer 1-like conformation is a precursor to the post-fusion conformation, trimer 1-specific antibodies could be protective by inhibiting membrane fusion. Compared to the trimer 2 conformation, one would expect that among antibodies generated against the trimer 1 conformation, a greater number would recognize epitopes in the S2 subunit. Most neutralizing antibodies that have been characterized, however, recognize epitopes in the S1 subunit, primarily in the RBD and NTD, and antibodies recognizing S2 epitopes are frequently weaker neutralizers than those recognizing S1 epitopes^36,37^. Olia et al. reported lower titers of neutralizing antibodies when mice were inoculated with “aged” spike preparations that would be expected to contain largely trimer 1-like conformations^29^. These observations suggest that constructs favoring trimer 2 under physiological conditions would be generally preferable for subunit vaccines. Interestingly, recent results from Sanofi/GSK showed that a Beta strain-based spike-foldon subunit vaccine (which, based on our results, should be predominantly trimer 2) used as a booster induces a better Omicron-cross reactive antibody response than a reference sequence-based mRNA vaccine^12^. The Omicron spike sequence contains numerous mutations not present in the Beta spike sequence, but based on our results, both favor the trimer 2 conformation.

The low-temperature and low-pH behavior of the soluble spike constructs is unlikely to be a critical factor for vaccines post-administration under physiological conditions but could be important to consider during manufacturing, formulation and storage of vaccines or diagnostic reagents. In particular, monitoring proportions of trimer 1 and 2 could be an informative quality control step to assess batch-to-batch variability during spike protein manufacturing. Selection of variants favoring trimer 2, when possible, could improve storage stability. Frozen storage has been shown to reduce the temperature related conformational sensitivities^28^. Formulation at low pH would favor the more compact trimer 2 conformation and would be expected to be more appropriate for long term storage, as we have observed over the course of 24 weeks.

Our results indicate that although spike constructs adopting the trimer 1 conformation (eg. reference strain spike fused to foldon) are stoichiometrically trimeric, the properties of their constituent protomers are similar to monomeric forms of the same proteins. Specifically, thermal stability (DSC), solvent accessibility (HDX) and certain antibody epitopes are similar for trimer 1 and monomeric species. It is worth considering whether vaccine antigens consisting of trimer 1 should be preferred over monomeric antigens given potential issues associated with the use of heterologous trimerization domains. In particular, the presence of existing antibodies (eg. antibodies against T4 phage proteins are prevalent in the human population^38^) could limit vaccine performance. Also, auto-antibodies could be induced if a human protein trimerization sequence is used, and the presence of immune-dominant epitopes in trimerization sequences may impair immune response to the spike sequence^39^.

In summary, starting with the observation of conspicuous double-trimer peaks by analytical SEC, we proceeded to characterize an array of soluble, recombinant SARS-CoV-2 spike constructs by multiple biophysical methods to understand better this apparent heterogeneity. Our results indicate that different spike variants can adopt two divergent trimer conformations: one corresponding to a conventional, compact, prefusion conformation, and a second with loosely-associated ectodomain protomers consistent with a recently-reported, alternative conformation^13^. For the development of new, cross-protective spike subunit vaccine antigens, the impact of variant-associated mutations on conformational preference should be carefully monitored.

### Limitations of the current study

A primary limitation of the current study is the lack of direct evidence comparing the effectiveness of spike preparations favoring trimer 1 *vs* trimer 2 as vaccine antigens in vivo*.* This type of analysis was not possible because different variants contain a variety of mutations that may impact function in other ways than any effect on conformation preference. Likewise, we have not found an effective method for purifying homogenous preparations of trimer 1 or trimer 2 (for variants that exhibit a mix of conformations), and in any case, dynamic interconversion between the two forms may reduce the utility of including such a step.

## Methods

### Protein production and purification

Spike proteins were produced in transiently or stably transfected CHO cells as described previously (^8^ and^18^ for transient and stable, respectively). CHO-DXB11 cells^40^ were provided by L. A. Chasin; CHO^55E1^™ and CHO^2353^™ cell lines were derived from CHO-DXB11 in-house as described^21,41^. The identities of these cell lines were previously verified by genome sequencing (data not shown). The amino acid sequences of the reference-strain constructs fused to resistin and foldon were as described^8^ but with C-terminal epitope tags (FLAG-His_6_) removed. Expression constructs encoding SARS-CoV-2 variant spike proteins, with mutations as detailed in Supplementary Table 1, were generated by mutagenesis or replacement of restriction fragments with synthetic fragments containing the required sequence modifications. All constructs contain prefusion-stabilizing 2P mutations^42^. Proteins were purified from CHO supernatants using a one-step affinity method (NGL COVID-19 Spike Protein Affinity Resin (Repligen)) or a multi-step non-affinity-based proprietary method. Immediately following purification, protein preparations were formulated by tangential flow filtration or desalting columns into DPBS (Gibco) adjusted with NaOH to pH 7.8 and aliquots were frozen at − 80 °C. For analysis at low pH samples were thawed and buffer exchanged into 20 mM acetate, 150 mM NaCl, pH 5.5 using 5 ml Zeba spin 7K MWCO desalting columns (Thermo Scientific) according to manufacturer’s instructions.

Purified dimeric V_H_H-Fcs were produced as described^16^. Monomeric V_H_Hs, enzymatically biotinylated at a single site in their C-terminal BAP tag, were also produced as described^16^ and further loaded onto a Superdex 75 10/300 GL column (Cytiva) connected to an ÄKTA FPLC protein purification system (Cytiva) that used PBS/5 mM EDTA as the running buffer. Eluted monomeric fractions were collected and used in the ELISA (see below).

### UPLC-SEC/MALS

SEC-MALS was performed on a Waters Acquity H-Class instrument using a Waters 4.6 × 150 mm BEH450 SEC column. The mobile phase was DPBS + 0.02% Tween 20, pH 7.2 or 50 mM acetate, 150 mM NaCl, pH 5. The flow rate was 0.4 ml/min. Sample load was 10 µg when possible or 10 µl when the protein concentration was less than 1 mg/ml. Detection was by absorbance at 280 nm and by MALS. MALS data was collected on a Wyatt microDAWN detector and was processed using Astra 8. In most cases the A_280_ signal was used as the concentration source with the E^0.1%^ calculated from the amino acid sequence. The 14- and 24-week time points included refractive index (RI) detection on a Wyatt Optilab UT-rEX detector. Molar masses were calculated in Astra 8 using both UV and RI signals as the concentration source. A significant difference was observed, so the protein conjugate analysis in Astra 8 was used assuming that the modifier was glycan with a dn/dc of 0.13. The resulting molar masses of the protein portion were within 2–10% of the calculated mass.

### DSC

DSC was performed on a Malvern microCal DSC system. With one exception samples were diluted to 0.4 mg/ml in DPBS, pH 7.8. The stock solution of the reference variant with no trimerization domain was 0.32 mg/ml and was analyzed without dilution. The temperature ramp ranged from 20 to 100 °C at a rate of 60 °C per hour. Buffer blanks were run between samples. Data were analyzed using Origin 7 software with manual baseline fitting.

### DSF

DSF was used to determine the melting curve of purified spike variants. All samples are formulated in DPBS at pH 7.8. The protein concentration was slightly different from one sample to another and ranged from 1 to 4 µM. A volume of 15 µl of each sample was placed in a 96-well qPCR plate (Applied Biosystems, Cat# N8010560) to which was added 5 µl of a Sypro Orange (Invitrogen, Cat# S6650), pre-diluted 125-fold from the manufacturer’s stock solution with DPBS. The samples were mixed by pipetting up and down after which the plate was sealed with an optical adhesive cover (Applied Biosystems, Cat# 4311971) and centrifuged for 5 min at 1000*g*. Thermal melt curves were recorded using the qPCR instrument QuantStudio7 with a thermal gradient ramp from 25 to 95 °C at 0.9 °C/min. Data was analyzed using the Protein Thermal Shift software (v1.4) from Thermo Fisher.

### ELISA

For ELISAs with V_H_H-Fcs, recombinant SARS-CoV-2 spike glycoprotein ectodomains were coated overnight at 4 °C onto NUNC Immulon 4 HBX microtiter plates (Thermo Fisher, Cat# 3855) at 50 ng/well in 100 µL of phosphate-buffered saline (PBS), pH 7.4. The next day, plates were blocked with 200 µl PBSC (1% (w/v) casein (Sigma, Cat# E3414) in PBS) for 1 h at room temperature, then washed five times with PBST (PBS supplemented with 0.05% (v/v) Tween 20) and incubated at room temperature for 1 h on rocking platform at 80 rpm with 100 µl of various concentrations of V_H_H-Fcs diluted in PBSTC (PBS/0.2% casein/0.1% Tween 20). Plates were washed five times with PBSTC and binding of V_H_H-Fcs was detected using 100 µl of 1 µg/ml horse radish peroxidase-conjugated goat anti-human IgG (Sigma, Cat# A0170). Finally, wells were washed 10 times and incubated with 100 µl peroxidase substrate solution (SeraCare, Cat# 50-76-00) at room temperature for 15 min. Reactions were stopped by adding 50 µl of 1 M H_2_SO_4_ to wells, and absorbance were subsequently measured at 450 nm using a Multiskan FC photometer (Thermo Fisher). The same steps were performed for ELISAs with monomeric V_H_Hs, except that biotinylated V_H_Hs were used instead of V_H_H-Fcs and the binding of the V_H_Hs was detected using horseradish peroxidase-conjugated-streptavidin (Jackson ImmunoResearch, Cat# 016-030-084).

### Analytical ultracentrifugation

Sedimentation velocity experiments were performed on a Beckman ProteomeLab-XLI instrument. Cells were assembled using sapphire windows and 2-sector charcoal filled Epon centerpieces with a 3 mm pathlength. Reference sector buffers were DPBS, pH 7.8 or 20 mM acetate, 150 mM NaCl, pH 5.5. Samples were temperature equilibrated in the instrument for at least 1.5 h before data collection. The temperature was 20 °C, the rotor speed was 35,000 rpm, and absorbance scans were collected every 4 min. Data were processed using Sedfit^43^ and peak integrations were done using Gussi^44^.

### Near and far UV circular dichroism

CD spectra were collected at 20 °C on a Jasco J-815 spectropolarimeter. Near UV spectra were collected from 340 to 250 nm at a scan rate of 50 nm/min, a data pitch of 0.5 nm, bandwidth of 0.5 nm, a response factor of 1 s, and 25 accumulations were collected. The sample volume was 1 ml and the cell pathlength was 1 cm. Samples were undiluted after thawing or after buffer exchange and all but three samples ranged from 0.8 to 1.3 mg/ml; the S(Delta+)-R, S(alpha-G614D)-R, and S(Delta)-F constructs ranged from 0.6 to 0.7 mg/ml. At pH 5.5 the samples were analyzed without dilution following the buffer exchange and concentrations ranged from 0.8 to 1.2 mg/ml for most variants, with the three aforementioned variants ranging from 0.5 to 0.6 mg/ml. For the far UV CD spectra the samples were diluted to 0.15 mg/ml and the cell pathlength was 1 mm. Far UV CD spectra were collected from 260 to 195 nm using the same scan rate, data pitch, bandwidth and number of accumulations used for the near UV CD spectra.

### Bottom-up hydrogen exchange mass spectrometry

Spike HDX-MS was performed as previously described^16^. Briefly, spike constructs were diluted to 8 μM in 10 mM Tris (pH 7.4). Labelling was initiated by a 10× dilution with 50% D_2_O (10 mM Tris, pD 7.0) at 20 °C in an automated fashion using an HDx-3 Pal (Trajan Scientific and Medical). The reaction was quenched after 0.5, 3 and 60 min with 2% formic acid (FA) (pH 2.2) at 4 °C, and 15 pmol was injected. On-line electrochemical reduction was achieved with a µ-Prepcell (Antec Scientific)^45^ followed by digestion with immobilized Nepenthesin-II (Affipro)^46^ and trapping with an ACQUITY UPLC BEH C18 Vanguard Pre-column (Waters, 130 Å, 1.7 µm, 2.1 mm × 5 mm) at 50 µL/min (0.23% FA) over 6 min. Peptides were separated with an ACQUITY UPLC BEH C18 (Waters, 130 Å, 1.7 µm, 1 × 100 mm) using a 2–40% gradient over 15 min (0.23% FA in acetonitrile). MS data was collected in triplicate using a Synapt G2-Si (Waters) over a 300–1600 m/z mass range. Peptide identifications were made by injecting undeuterated samples analyzed with data-dependent acquisition followed by database searching with Mascot. Deuteration was assigned with MS Studio^47^, and significant changes were assigned based on a pooled two-state student T-Test performed for each state and time point (ΔD > 3 × standard deviation, 1-p value > 0.98).

### Ethics declaration

No human or animal subjects were used in the experiments described in this manuscript.

### Supplementary Information

Supplementary Information 1.

Supplementary Information 2.

Supplementary Information 3.

Supplementary Information 4.

## Data Availability

The datasets generated during and/or analysed during the current study (eg. raw data used to generate figures) are available from the corresponding author on reasonable request.
