# Supplementary material for: Influence of variant-specific mutations, temperature and pH on conformations of a large set of SARS-CoV-2 spike trimer vaccine antigen candidates
Source: Sci Rep. 2023 Oct 1;13:16498. doi: 10.1038/s41598-023-43661-2 (PMC10543594; doi:10.1038/s41598-023-43661-2)
Supplement: Supplementary file 1 — Supplementary Information 1. [file 41598_2023_43661_MOESM1_ESM.pdf]

Full name of construct	Abbreviation	Mutations[a,b]
Spike (reference strain)-resistin fusion	S(Ref)-R	none
Spike (reference strain)-foldon fusion	S(Ref)-F	none
Spike (reference strain + D614G)-resistin fusion	S(Ref-D614G)-R	D614G
Spike (reference strain + D614G)-foldon fusion	S(Ref-D614G)-F	D614G
Spike (Alpha variant)-resistin fusion	S(Alpha)-R	69del, 70del, 144del, N501Y, A570D, D614G, P681H, T716I, S982A, D1118H
Spike (Alpha G614D)-resistin fusion	S(Alpha-G614D)-R	69del, 70del, 144del, N501Y, A570D, P681H, T716I, S982A, D1118H
Spike (Gamma variant)-resistin fusion	S(Gamma)-R	L18F, T20N, P26S, D138Y, R190S, K417T, E484K, N501Y, D614G, H655Y, T1027I
Spike (Gamma variant)-foldon fusion	S(Gamma)-F	L18F, T20N, P26S, D138Y, R190S, K417T, E484K, N501Y, D614G, H655Y, T1027I
Spike (Beta variant)-resistin fusion	S(Beta)-R	D80A, D215G, 241del, 242del, 243del, K417N, E484K, N501Y, D614G, A701V
Spike (Beta variant)-foldon fusion	S(Beta)-F	D80A, D215G, 241del, 242del, 243del, K417N, E484K, N501Y, D614G, A701V
Spike (Kappa variant)-resistin fusion	S(Kappa)-R	G142D, E154K, L452R, E484Q, D614G, P681R, Q1071H
Spike (Delta variant)-resistin fusion	S(Delta)-R	T19R, G142D, E156del, F157del, R158G, L452R, T478K, D614G, P681R, D950N
Spike (Delta variant)-foldon fusion	S(Delta)-F	T19R, G142D, E156del, F157del, R158G, L452R, T478K, D614G, P681R, D950N
Spike (Delta-plus variant)-resistin fusion	S(Delta+)-R	T19R, G142D, E156del, F157del, R158G, K417N, L452R, T478K, D614G, P681R, D950N
Spike (Omicron BA.1 variant)-resistin fusion	S(BA1)-R	A67V, 69-70del, T95I, GVYY142-145D, NL211-212I, ins214EPE, G339D, S371L, S373P, S375F, K417N, N440K, G446S, S477N, T478K, E484A, Q493R, G496S, Q498R, N501Y, Y505H, T547K, D614G, H655Y, N679K, P681H, N764K, D796Y, N856K, Q954H, N969K, L981F
Spike (Hexapro)-resistin fusion	S(Hexa)-R	D614G, F817P, A892P, A899P, A942P
Spike (Hexapro)-foldon fusion	S(Hexa)-F	D614G, F817P, A892P, A899P, A942P
Spike (reference strain) without trimerization domain	S(Ref)-noTD	none
Spike (Delta variant) without trimerization domain	S(Delta)-noTD	T19R, G142D, E156del, F157del, R158G, L452R, T478K, D614G, P681R, D950N

Supplementary Table 1: Details of spike constructs used in the current study. [a] All constructs contain mutations to abrogate furin site (RRAR->GGAS, 682-685) as well as prefusion-stabilizing '2P' (KV->PP, 986-987) mutations. [b] Amino acid numbering and mutations are relative to reference strain sequence.

	DPBS pH 7.8						Acetate pH 5.5						DPBS pH 7.8	Acetate pH 5.5	
	Hexamer		Trimer 1		Trimer 2		Hexamer		Trimer 1		Trimer 2				
**Construct**	ret time (min)	% peak area	ret time (min)	% peak area	ret time (min)	% peak area	ret time (min)	% peak area	ret time (min)	% peak area	ret time (min)	% peak area	$S_{20,w}$	$S_{20,w}$	ΔCD
S(Ref)-R	2.946	1.9	3.272	85.9	3.52	11.2	3.01	3.95			3.465	91.39	12.66	14.62	11.9
	2.948	1.84	3.275	84.8	3.52	12.2	3.004	4.2			3.465	94.71		14.65	
S(Beta)-R	3.007	6.46	3.291	21.85	3.535	69.45	2.979	5.58			3.446	94.42	14.03	14.51	2.6
	3.009	6.43	3.294	21.58	3.535	69.49	2.983	5.52			3.446	94.48			
S(Gamma)-R	2.958	1.32	3.348	94.91			2.982	6.62			3.467	88.84	12.86	14.58	11.1
	2.974	1.31	3.349	95.23			2.983	6.65			3.467	89.1			
S(Kappa)-R	3.028	10.41	3.373	23.85	3.565	59.17	3.029	8.57			3.493	84.49	13.64	14.48	6.3
	3.035	10.25	3.372	23.57	3.566	59.83	3.024	9.52			3.493	82.96			
S(Delta)-R	3.042	17.44	3.361	15.76	3.566	64.43	3.054	14.43			3.51	85.57	13.67	14.24	5.0
	3.044	16.63	3.354	16.63	3.566	64.48	3.05	15.35			3.51	84.65			
S(Delta+)-R	3.052	27.92	3.359	13.83	3.595	53.55	3.033	26.53			3.516	66.96	13.78	14.41	2.5
	3.052	27.66	3.358	13.95	3.596	54.88	3.034	25.51			3.515	66.99			
S(BA1)-R	2.979	3.69			3.508	91.16	2.944	1.89			3.433	94.25	14.38	14.48	1.4
	2.987	3.86			3.512	91.68	2.944	2.36			3.434	93.23			
S(Alpha)-R	2.997	13.82	3.32	28.7	3.534	54.18	2.989	8.91			3.462	87.97			
	2.998	13.94	3.32	28.7	3.533	54.24	2.987	9.06			3.463	90.94			
S(Ref-D614G)-R	2.969	0.69	3.33	33.49	3.532	63.02					3.47	97.49	12.86	14.41	7.2
	2.97	0.69	3.336	33.56	3.531	62.77					3.47	91.16			
S(Alpha-G614D)-R	2.94	2.22	3.348	87.4							3.488	82.22	12.97	14.49	ND
	2.945	2.27	3.352	87.48							3.489	81.88			
S(Hexa)-R	3.077	8.88	3.349	3.33	3.586	83.07	3.039	9.63			3.494	82.87	14.21	14.24	2.7
	3.077	8.67	3.355	3.61	3.585	83.15	3.034	9.66			3.495	82.6		14.28	

Supplementary Table 2: Summary of SEC, SV-AUC and CD results for resistin constructs. Retention times and % peak areas are shown for samples in both DPBS and acetate buffers, as are the $S_{20,w}$ values determined by sedimentation velocity (AUC). Calculated delta (Δ) CD values correspond to rms differences between near UV CD spectra obtained in DPBS (pH 7.8) and acetate (pH 5.5) buffers. For tests performed in duplicate, individual measurements are shown. For SEC results, empty cells indicate that a given peak (eg. trimer 1 at pH 5.5) was not observed.

	DPBS pH 7.8						Acetate pH 5.5						DPBS pH 7.8	Acetate pH 5.5	
	Hexamer		Trimer 1		Trimer 2		Hexamer		Trimer 1		Trimer 2				
**Construct**	ret time (min)	% peak area	ret time (min)	% peak area	ret time (min)	% peak area	ret time (min)	% peak area	ret time (min)	% peak area	ret time (min)	% peak area	$S_{20,w}$	$S_{20,w}$	ΔCD
S(Ref)-F	3.007	3.61	3.407	92.71			3.046	1.11			3.528	91.94	12.39	14.32	14.1
	3.006	3.52	3.407	92.85			3.013	1.1			3.528	92.09			
S(Beta)-F	3.01	1.55	3.347	27.83	3.522	70.16					3.462	97.25			
	3.014	1.54	3.347	27.84	3.523	70.2					3.462	97.18			
S(Gamma)-F			3.462	98.49							3.513	99.57	12.59	14.21	9.5
			3.461	98.43							3.514	99.47			
S(Delta)-F	3.028	7.38	3.415	49.44	3.548	39.23	3.065	4.16			3.543	92.02	12.65	14.29	7.9
	3.025	7.39	3.415	49.81	3.548	39.04	3.091	3.48			3.548	92.03			
S(Ref-D614G)-F	3.03	5.77	3.438	37.08	3.569	53.11					3.529	89.28	12.86	14.23	6.1
	3.022	5.8	3.438	37.74	3.57	52.49					3.529	89.59			
S(Hexa)-F	3.129	5.41			3.595	93.16					3.539	98.88	13.66	13.888	2.8

Supplementary Table 3: Summary of SEC, SV-AUC and CD results for foldon constructs. Retention times and % peak areas are shown for samples in both DPBS and acetate buffers, as are the S20,w values determined by sedimentation velocity (AUC). Calculated delta (Δ) CD values correspond to rms differences between near UV CD spectra obtained in DPBS (pH 7.8) and acetate (pH 5.5) buffers. For tests performed in duplicate, individual measurements are shown. For SEC results, empty cells indicate that a given peak (eg. trimer 1 at pH 5.5) was not observed.

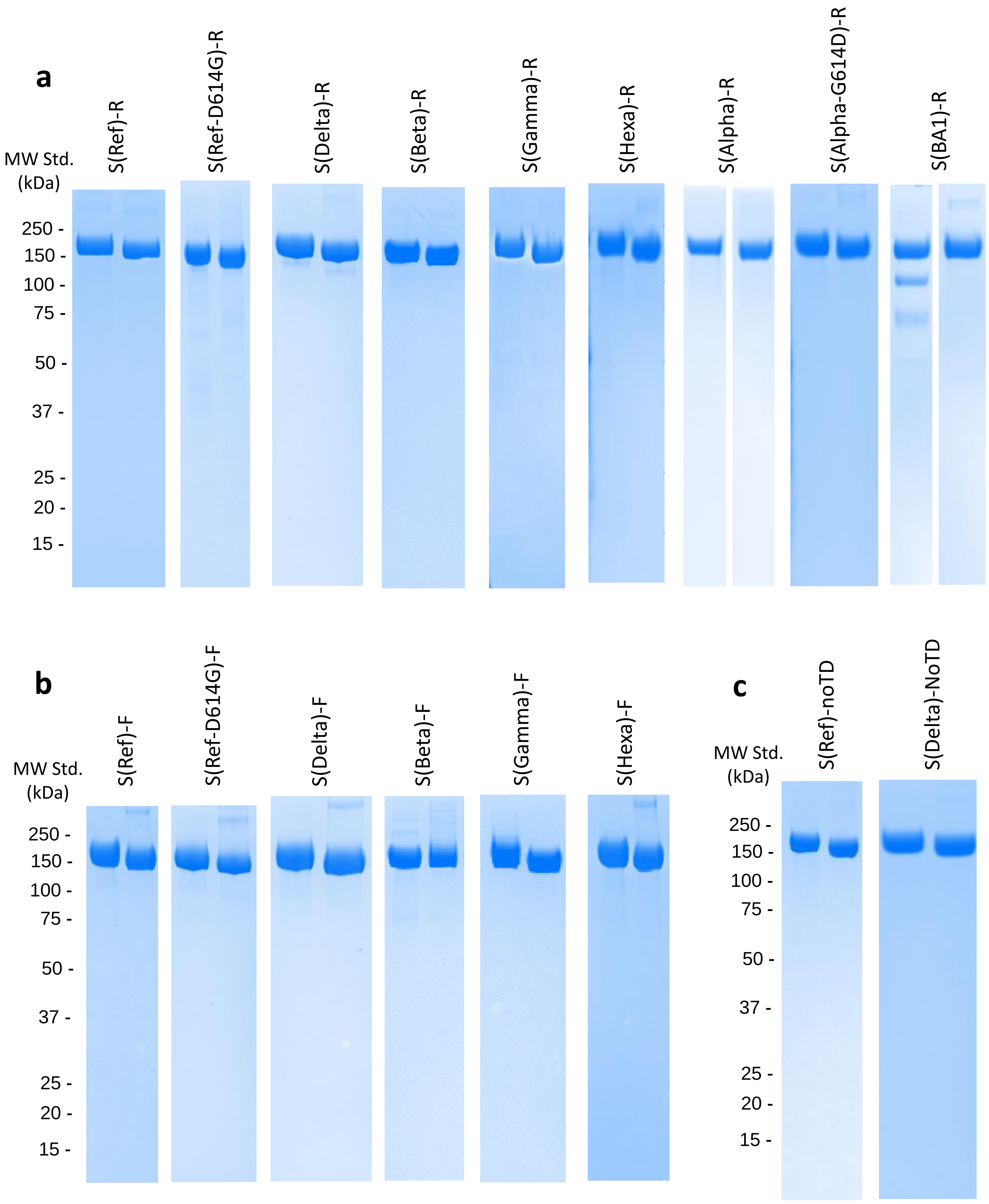

Supplementary Figure 1: SDS-PAGE/Coomassie staining of spike protein preparations. Two or three micrograms of purified resistin fusions (**a**)**,** foldon fusions (**b**) and constructs without heterologous trimerization sequences (**c**) were separated by SDS-PAGE under reducing (left lane) or non-reducing (right lane) conditions and stained with Coomassie Blue. For (S(Alpha)-R the lanes shown were part of the same gel, but intervening, non-relevant sample lanes were removed. For S(BA1)-R, the lanes shown are from two separate gels processed at the same time.

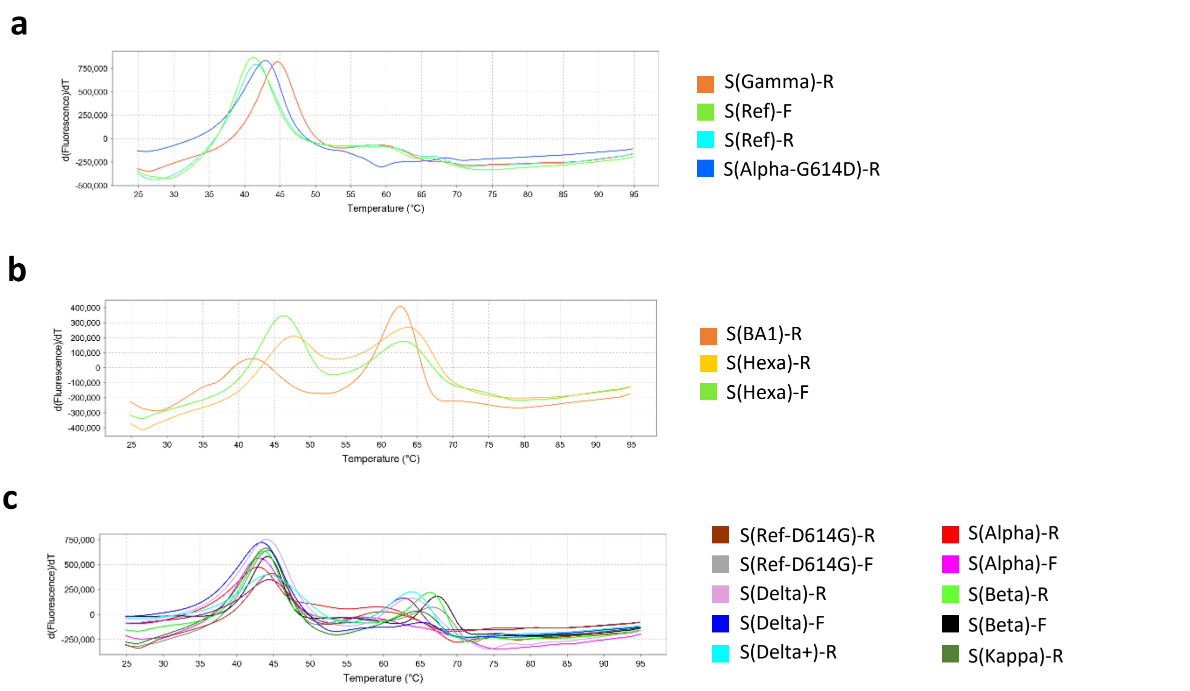

Supplementary Figure 2: Differential scanning fluorimetry melting curves. First-derivative of DSF data obtained in DPBS at pH 7.8 for (**a**) spike variants favoring trimer 1 at pH 7.8, (**b**) spike variants favoring trimer 2 at pH 7.8 and (**c**) spike variants with a mix of trimer 1 and trimer 2 at pH 7.8.

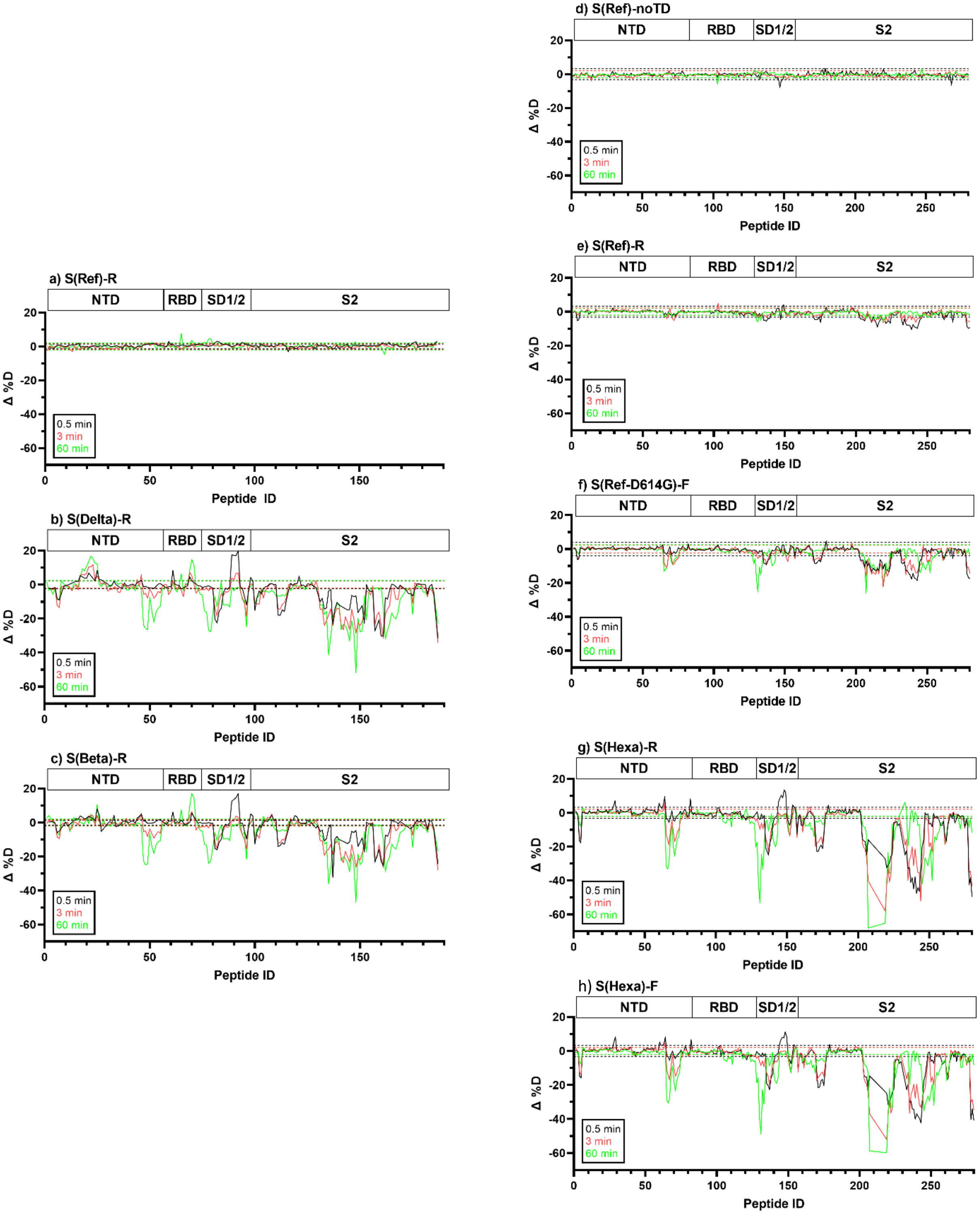

Supplementary Figure 3. HDX-MS Woods plots. Differential HDX (Δ %D) relative to S(Ref)-F is plotted as a function of peptide ID (see Supplementary Table HDX-S2 for a full list of peptides). Data collected in triplicate. Dashed lines represent ± 3 x pooled SD for each time point. (**a-c**) and (**d-g**) were collected in separated experiments; note that the number of peptides analyzed and the distribution of these peptides along the spike sequence were different for the two experiments. Δ %D measurements outside the dashed lines demonstrate either significantly reduced exchange (- Δ %D) or increased exchange (+ Δ %D) based on a 1-p value of 0.98. Key structural domains are indicated above the Woods plots.

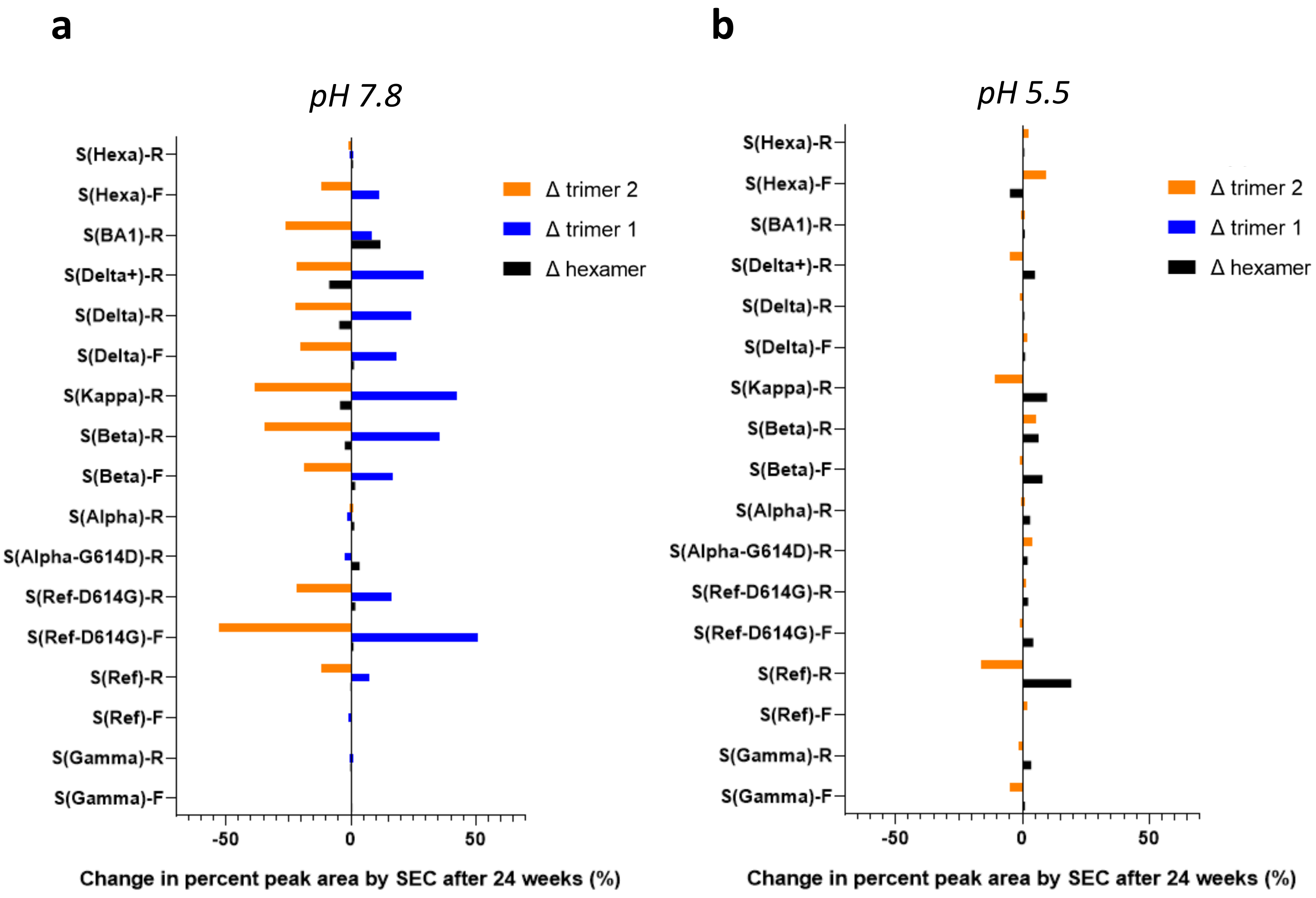

Supplementary Figure 4: Effects of long-term storage at 4°C at pH 7.8 or pH 5.5 on distribution of SEC peak areas. Changes in peak distribution after 24 weeks of storage at either pH 7.8 (**a**) or pH 5.5 (**b**). For constructs consisting mostly of trimer 1 at pH 7.8 (reference strain and Gamma variants), there was little change upon long term storage at 4°C. For constructs containing significant amounts of trimer 2 at pH 7.8, the proportion of trimer 2 decreased with prolonged storage at this pH. Since all constructs were predominantly trimer 2 at pH 5.5, there was little change in trimer distribution on long term storage at 4°C at this pH, although hexamer content increased for some variants.

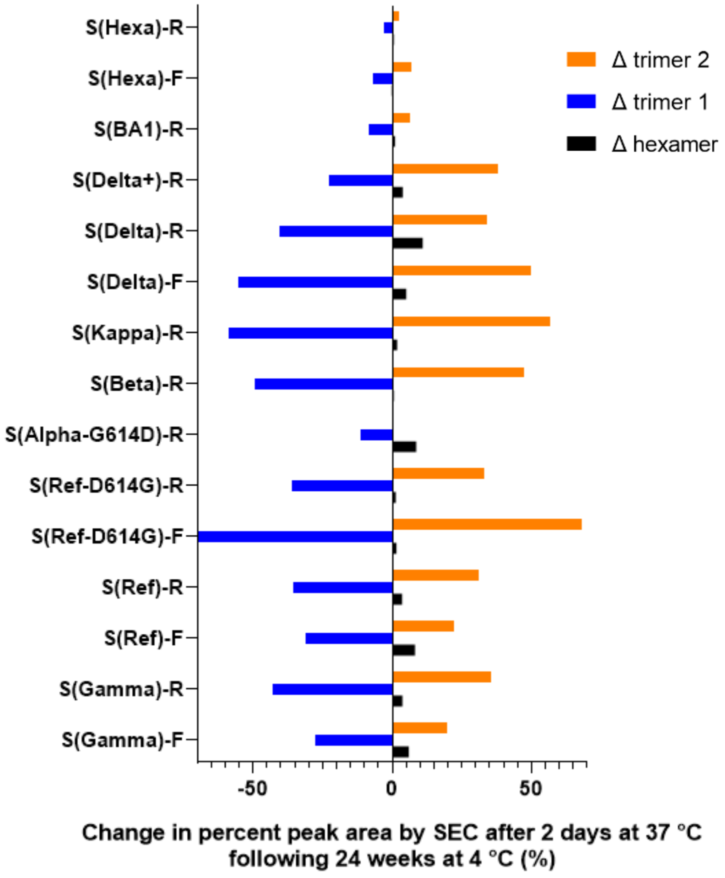

Supplementary Figure 5: Effect of long-term storage at pH 7.8 (4°C) is partially reversed with subsequent incubation at 37°C for 2 days. Spike preparations at pH 7.8 were stored at 4°C for 24 weeks then at 37°C for 2 days. Changes in SEC peak distributions occurring during the 37°C incubation were calculated from samples analyzed before and after the incubation period.

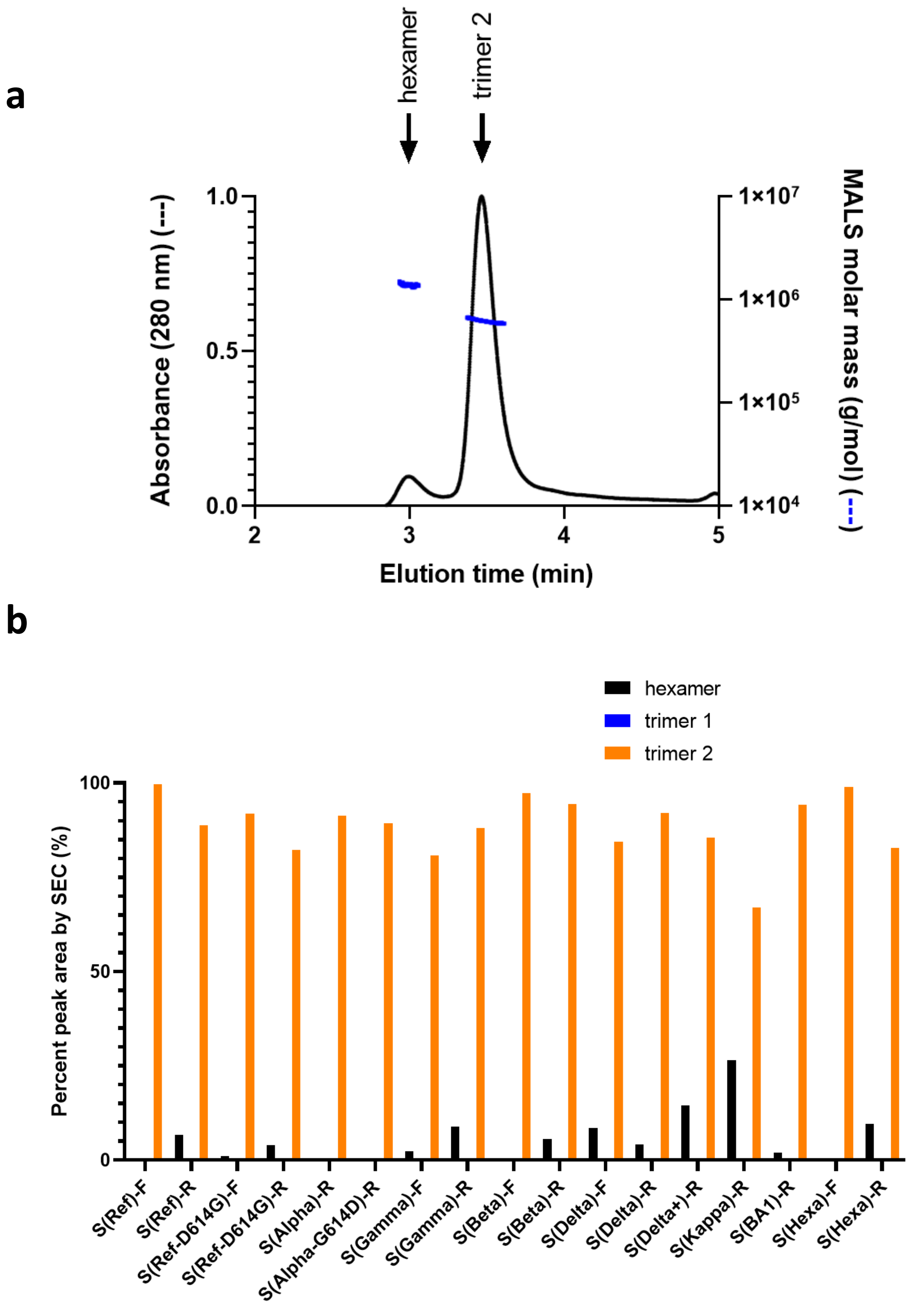

Supplementary Figure 6: At pH 5.5, SARS-CoV-2 spike protein variants display primarily a single trimer peak by analytical size exclusion chromatography (SEC). **a** Example of an A_{280} SEC profile of Alpha variant spike ectodomain fused to resistin (S(Alpha)-R) at pH 5.5, showing peaks with molar masses consistent with hexamer and trimer (trimer 2) species. **b** Distribution of trimer and hexamer SEC peaks for a selection of spike variants in DPBS at pH 7.8.

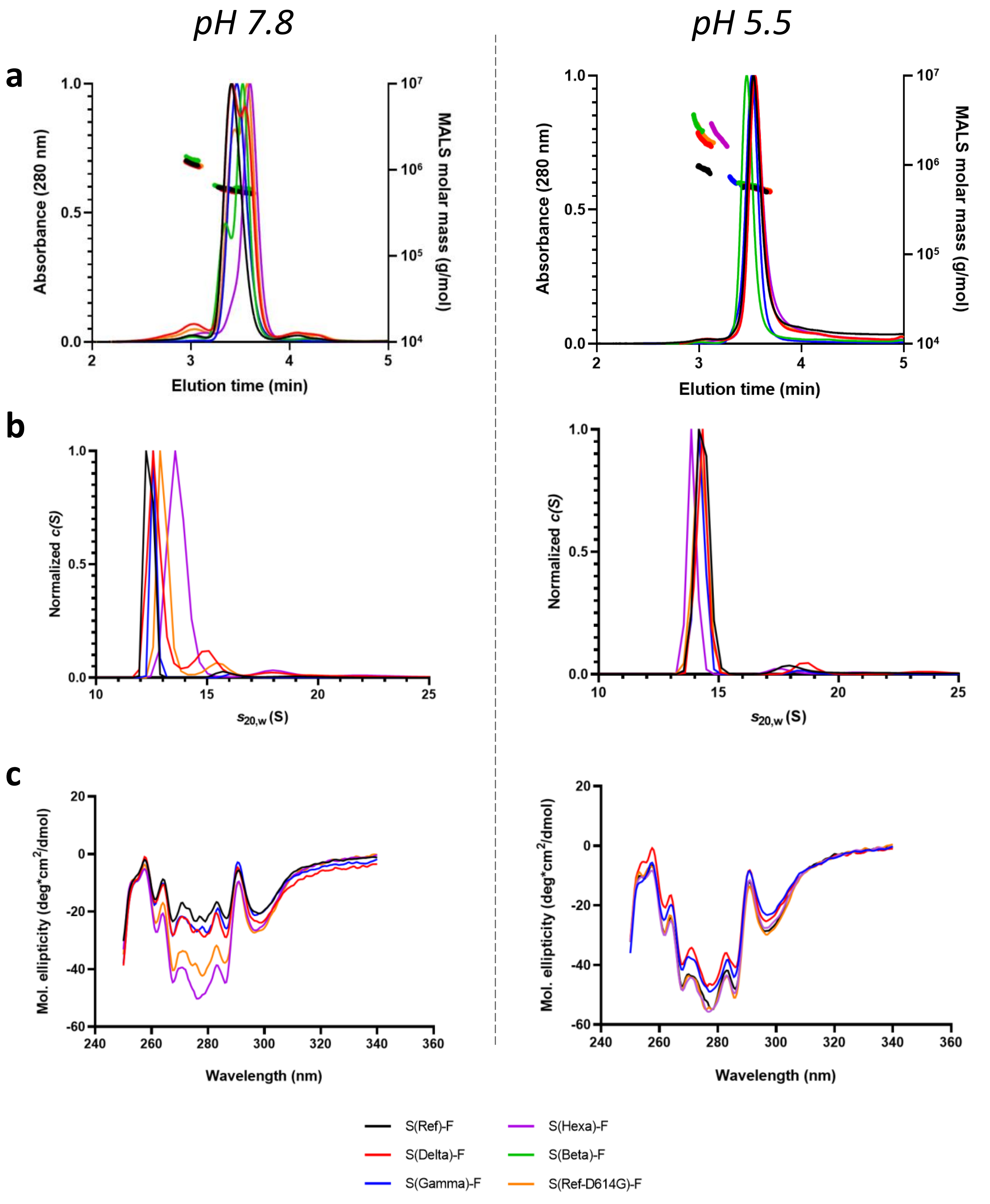

Supplementary Figure 7: Conformational variability of spike-foldon fusion constructs is reduced at low pH. UPLC-SEC (**a**), SV-AUC (**b**) and near-UV CD (**c**) demonstrate that conformational variability observed at pH 7.8 is greatly reduced at pH 5.5. All data shown is for foldon fusion constructs.

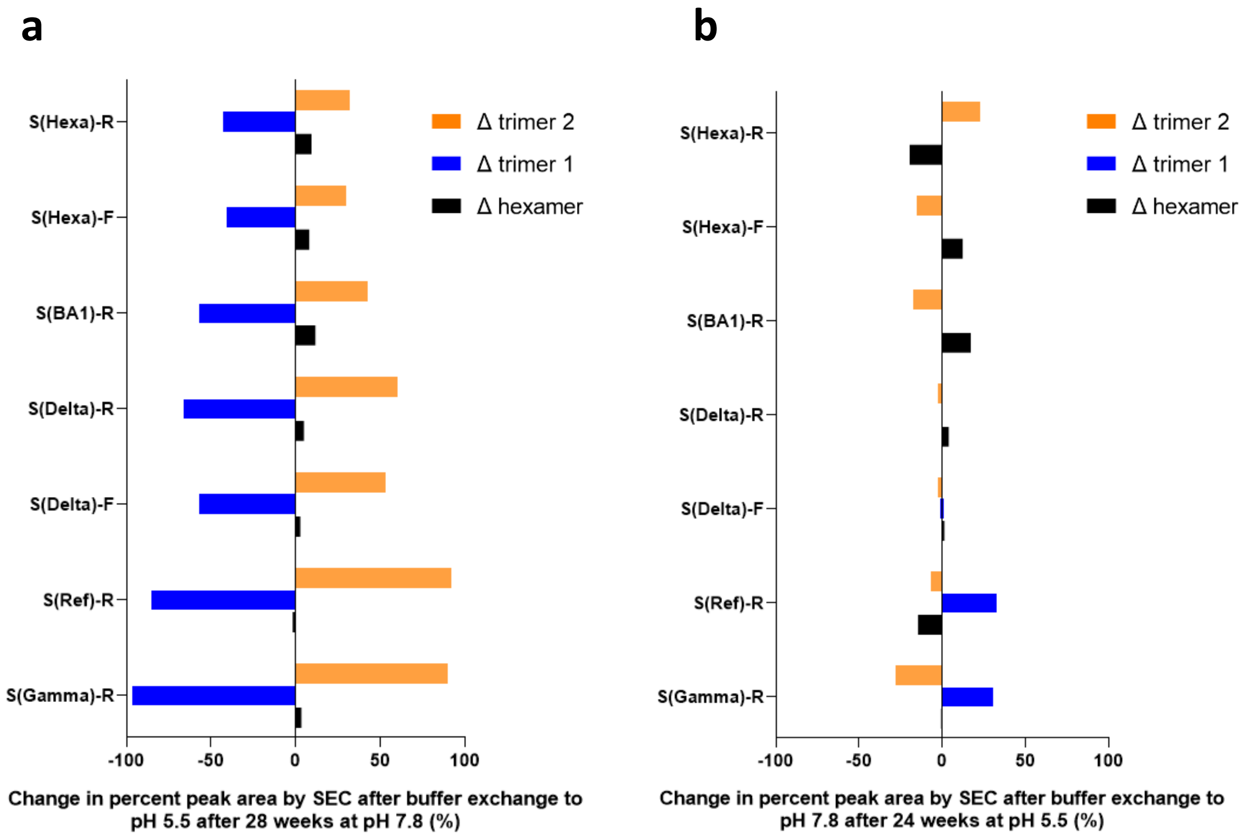

Supplementary Figure 8: Effects of long-term storage at pH 5.5 or 7.8 (4°C) are reversed to different extents by subsequent buffer exchange and 2-day incubation at the other pH. Following storage at 4°C for 24 weeks, spike preparations at pH 7.8 (**a**) and 5.5 (**b**) were buffer-exchanged to pH 5.5 and 7.8, respectively, and incubated at 4°C for 2 days. Changes in SEC peak distributions are shown comparing samples before buffer exchange to samples after buffer exchange and 2-day incubation at the final pH.

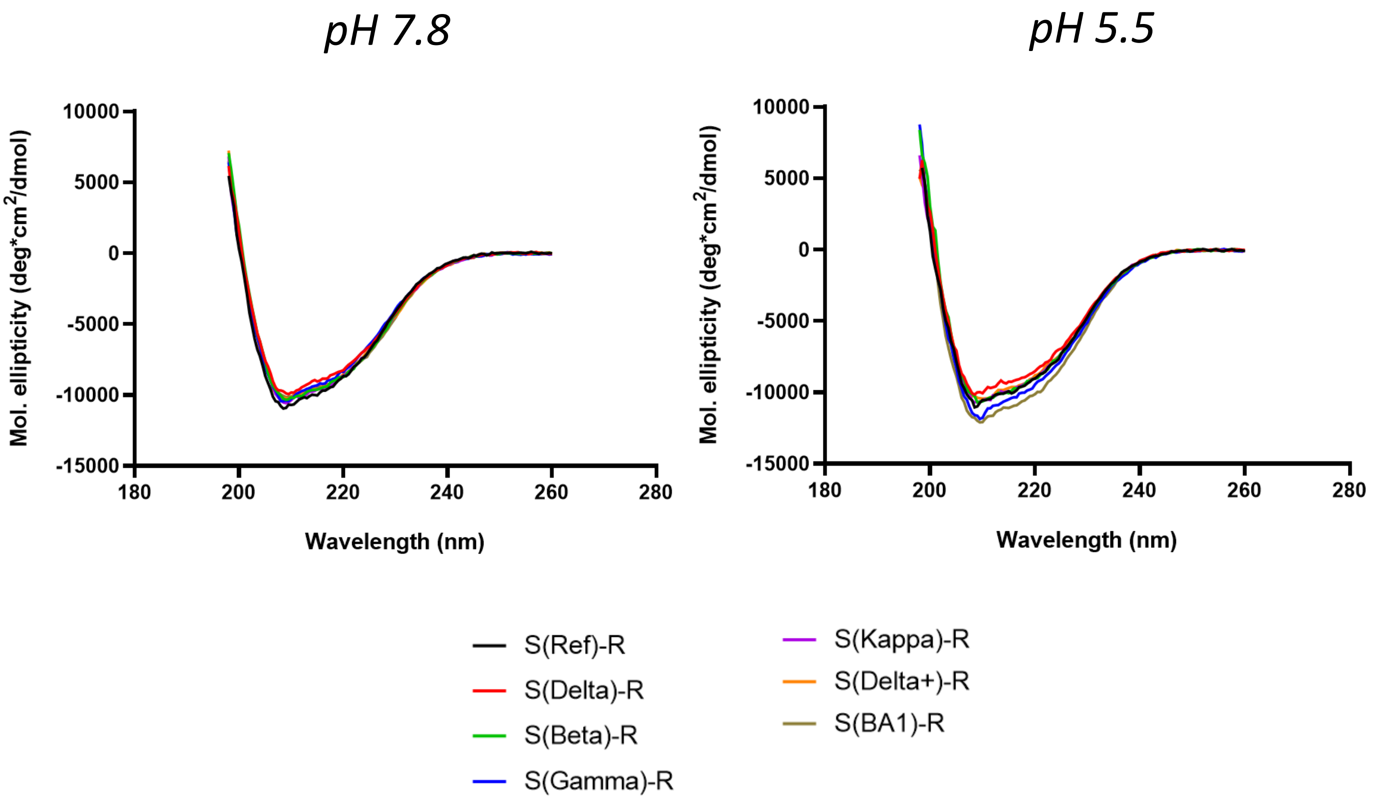

Supplementary Figure 9: Spike-resistin fusion constructs show similar far-UV CD spectra at pH 7.8 and 5.5. Compared to near-UV CD spectra which show significant differences between spike variants at pH 7.8, far-UV CD spectra are similar for all tested variants at pH 7.8 and 5.5.
