# Supplementary material for: Influence of variant-specific mutations, temperature and pH on conformations of a large set of SARS-CoV-2 spike trimer vaccine antigen candidates
Source: Sci Rep. 2023 Oct 1;13:16498. doi: 10.1038/s41598-023-43661-2 (PMC10543594; doi:10.1038/s41598-023-43661-2)

S(Ref)-R

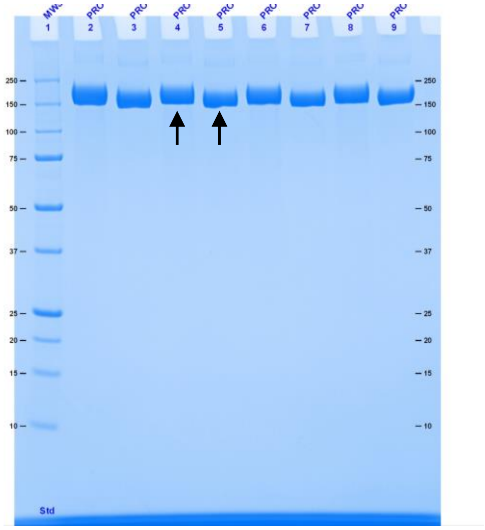

S(Ref-D614G)-R

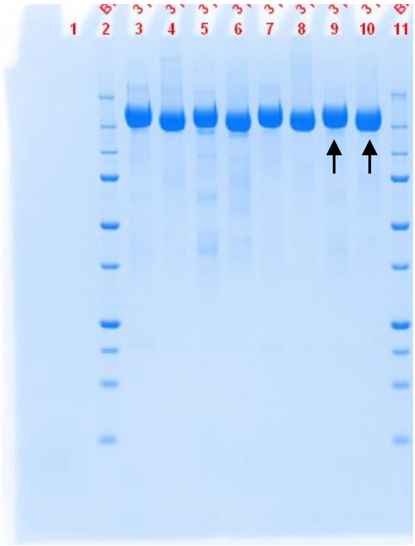

S(Delta)-R

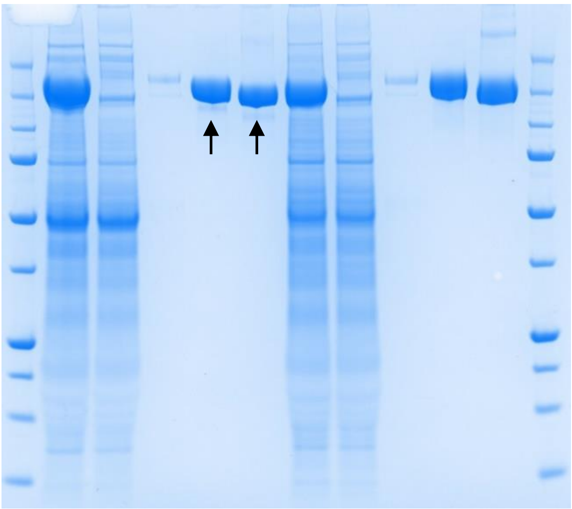

S(Beta)-R

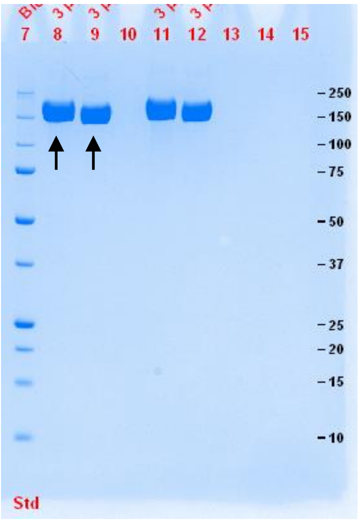

S(Gamma)-R

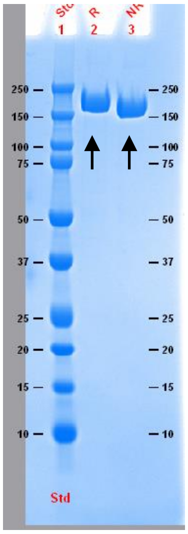

S(Hexa)-R

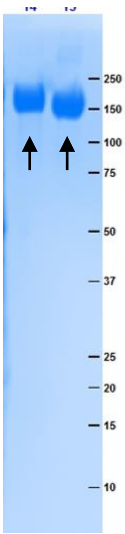

S(Alpha)-R

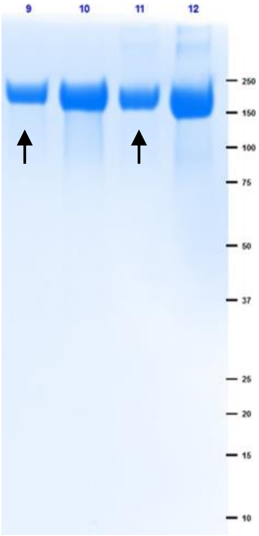

S(Alpha-G614D)-R

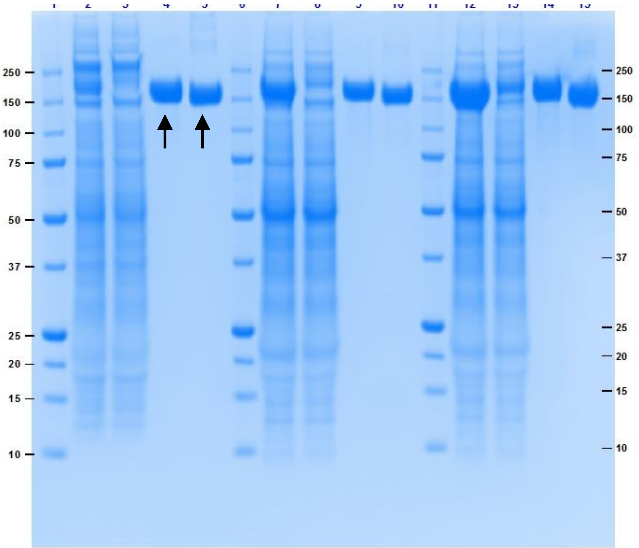

S(BA1)-R (reduced)

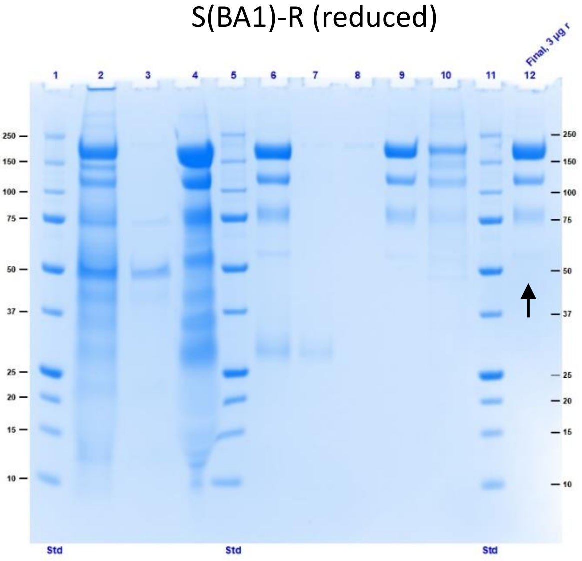

S(BA1)-R (non-reduced)

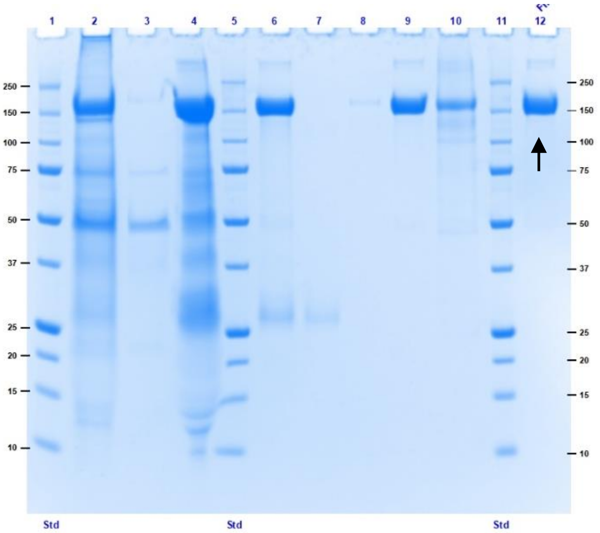

S(Ref)-F (lanes flipped horizontally in Supp Figure 1)

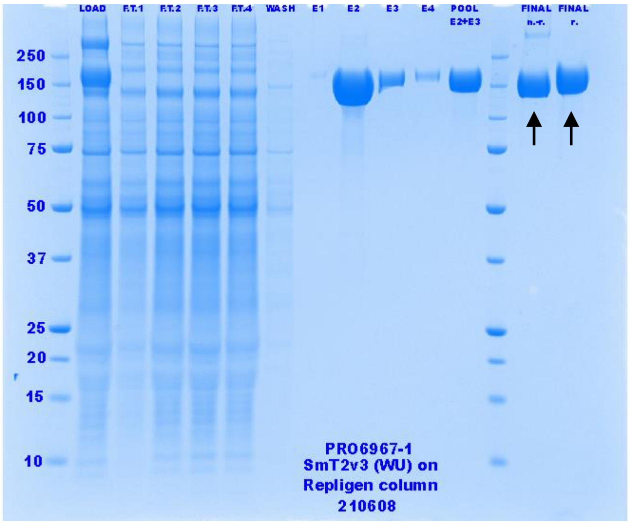

S(Ref-D614G)-F

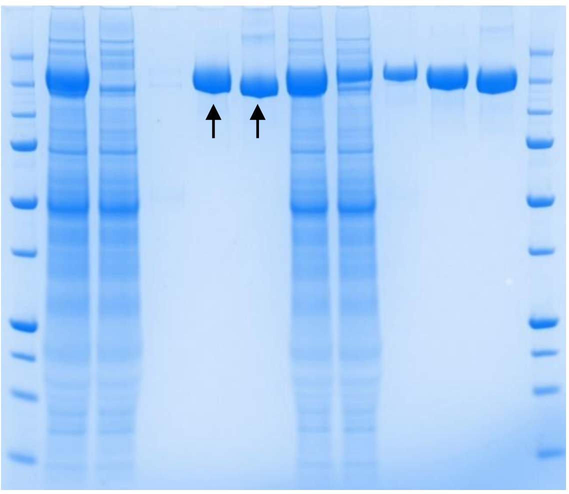

S(Delta)-F

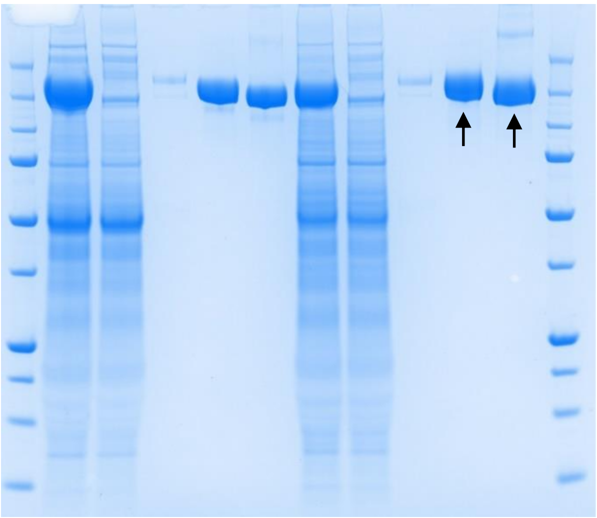

S(Beta)-F

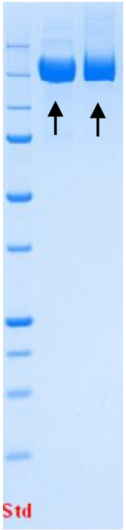

S(Gamma)-F

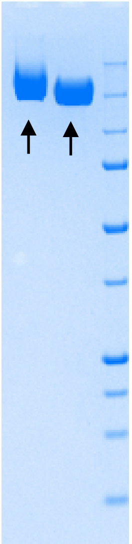

S(Hexa)-F

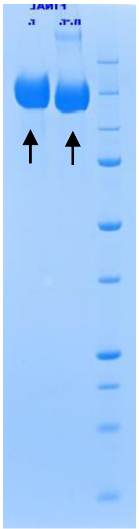

S(Ref)-noTD
(lanes flipped horizontally in Supp Figure 1)

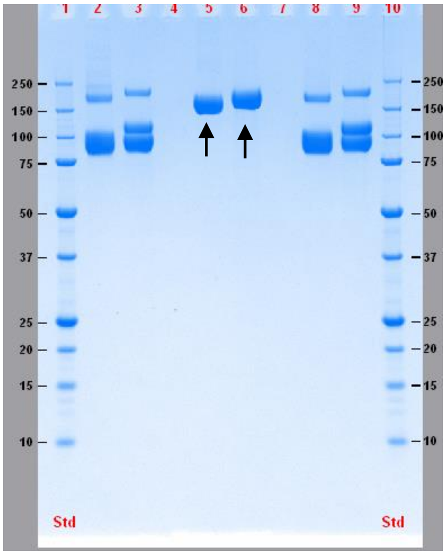

S(Delta)-noTD

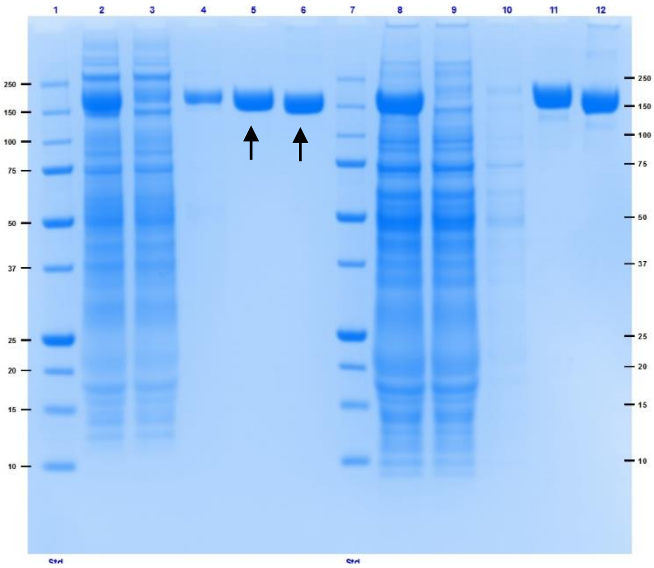

Supplement: Supplementary file 2 — Supplementary Information 2. [file 41598_2023_43661_MOESM2_ESM.pdf]
